# Home Educating in an Extended Family Culture and Aging Society May Fare Best during a Pandemic

**DOI:** 10.1371/journal.pone.0007221

**Published:** 2009-09-28

**Authors:** Wayne Dawson, Kenji Yamamoto

**Affiliations:** Research Institute, International Medical Center of Japan, Shinjuku-ku, Tokyo, Japan; University of Nottingham, United Kingdom

## Abstract

Large cities can contain populations that move rapidly from one section to another in an efficient transportation network. An emerging air-borne or contact based pathogen could use these transportation routes to rapidly spread an infection throughout an entire population in a short time. Further, in many developed countries, the aging population is increasing. The family structure in these societies may also affect the course of a disease. To help understand the impact of an epidemic on family structure in a networked population, an individual based computer model that randomly generates networked cities with a specified range of population and disease characteristics and individual schedules, infectivity, transmission and hygiene factors was developed. Several salient issues emerged. First, a city of highly active individuals may in fact diminish the number of fatalities because the average duration of the interactions between agents is reduced. Second, home schooling can significantly improve survival because the institutional clustering of weak individuals is minimized. Third, the worst scenario for an aging population is the nuclear family where the aged population is confined to large housing facilities. Naturally, hygiene is the first barrier to infection. The results suggest that societies where extended families and small groups manage most of their own affairs may also be the most suitable for defense against a pandemic. This may prove applicable in city planning and policy making.

## Introduction

Modern cities often have high speed transportation networks that permit individuals from distant sectors to come into contact in fairly short time intervals [1], [2], somewhat resembling a free scale network [3]. Some cities, particularly in Asia, also have increasing aging populations and a decreasing population of youth [4]–[8]. An emerging air-borne (or otherwise contact dependent) pathogen is likely to tap into these networks and rapidly spread through the population [9]–[12]. The particular infection profile [11], [13] of a given disease can vary with location, societal structure, habits, customs, etc. Whereas these are difficult to know beforehand, diseases preferentially attack the weakest members of a population, typically the young and the old [14], [15].

In developing a policy for defense against emerging infectious diseases (EIDs) [10], computer modeling is the first and safest step to take in testing what conditions may play the largest role in minimizing risks in a given population [1], [2], [9], [10]–[12]. The character of the model is important to interpreting the results and all such models are expected to have their limitations. As a whole, individual based models of disease transmission and infection offer the clearest similarity to a true human society [2]. Network type models help with describing generalized parameters [3]. However, it is difficult to model in the specific complexities of daily activities in the population. Individual based models can explicitly develop these networks with more characteristic features of real human populations.

We have developed a largely individual based model where the social networks and layout of the city are randomly constructed from a user defined population distribution. The population is highly networked to reflect some of the character of a modern city transportation network [1], [14], [16] with an infectious agent of variable infection profile and transmissibility. The detailed network relationships between individuals are explicitly included as well as the day to day activities of each individual. Thus, the model is a blend of both the individual based model combined with some of the general features of a network model.

The purpose of this study is to find out if family structure can change the outcome of a pandemic in any significant way and particularly in an aging population. Fatalities are generally discussed in terms of overall body counts where little attention is paid to how the societal structure can change that distribution or reduce the risks to different age groups. Over all, according to this study, the best policies should aim at distributed social structures. In addition, the model suggests that an extended family society that educates children at home appeared to fare significantly better at surviving an epidemic.

## Methods

### The Model and Simulation Methods

In this Section, we first describe the general features of the model and postpone the intricate details to the latter part of this main Section. There are many layers to the model and we found a top down approach seems the clearest way to understand this model without becoming mired in the details.

### Construction of the physical layout

The model assumes a city where transportation from any one point to another is sufficiently complete that interactions between individuals are not constrained by distance. In this respect, the model represents a network [3]. However, the model is also worked out from the daily activities of individuals and, in this sense, represents an individual based model. In this study, the particular city layouts were generated by a random assignment; though one could, in principle, enter all the attributes of the city manually.

Units of distance, which are used to build the city layouts and determine the infection probability between two individuals, were based on the likely range that an average sedentary person travels on foot under normal circumstances during a typical eight hour period. This varies considerably in different societies and environments, but is arbitrarily set to 100 m in this model. The distance was fixed in order to (1) obtain a fixed density, (2) reduce density dependent fluctuations and (3) permit examination of the scalability of the calculations. Using this fixed population density as a common standard, the actual range of movement of the individuals in a particular society was scaled to a larger or smaller value by adjusting the “temperature” (*T*) or degree of activity for the city independent of the average population density (the parameter *T* will be discussed later).

The area of the city is determined from the square of the range (100 m) times the number of individuals in the city. The locations for living quarters, school and work are generated randomly inside a square grid that is consistent with the above population density. Hence, the distance between neighbors is a distribution but the average density is equal for any given city of a given population size.

### Construction of the family structure and population distribution

Age groups in this model are divided into three categories: young (age 0 to 18), adult (age 19 to 55 years) and elderly-retired (E/R; age 55 to 100).

**Figure 1:**
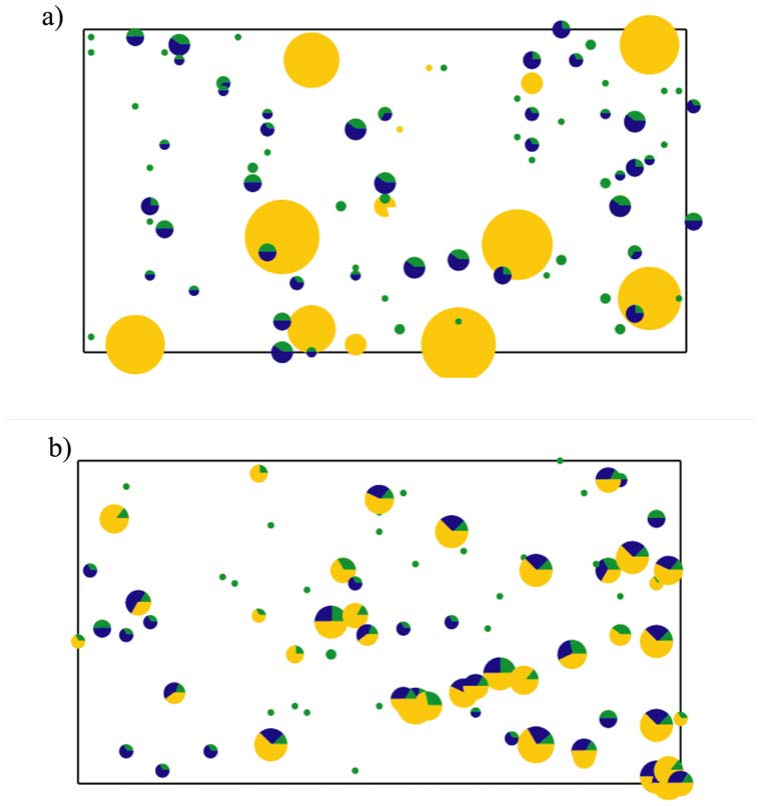
Examples of the initial conditions of the networked city. The size of the dots reflect the number of people within a household or a home for the elderly. The color of the dots reflects the age group involved: blue for youth 0–18 yrs, green for adults 19–55 yrs, and yellow represents the mature population 55+ years. (a) An example of a city containing nuclear families where the aged population is housed in large group facilities. (b) A similar example of a city containing extended families where the aged population stays with the family.

The constructed cities are built with two different types of social structures: nuclear families where the elderly and retired live separately in groups (Figure 1a) or extended families where all age groups live together as a family (Figure 1b). This is a predefined option at the initial construction. There is currently no mixing of these two types of systems. The radii of the circles shown in Figure 1 indicate the number of people clustered in the same location and group. The colors indicate the age groups: blue for children, green for adults and yellow for elderly/retired (E/R) individuals (Figs. 1).

**Figure 2:**
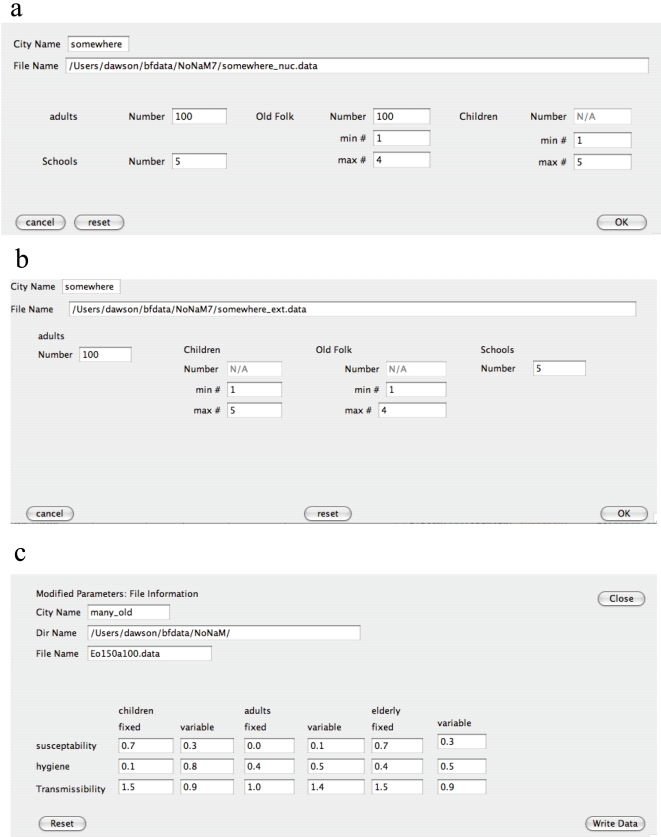
Example of the option menus used to build the cities and to establish infection profiles. (a) Option menu for building nuclear families. (b) Option menu for extended families. (c) Option menu for adjusting the infection profile of the individuals.

For both societies, the number of adults in the city is selected by the user who builds the city (Figure 2). The particular marital status (or habitation) of a given adult is decided automatically by a random number generator during this step.

If the adult is married, then it is randomly decided whether the adult (or adults) of the given family have children or not. If children are to be added, the numbers of children in each household is assigned randomly to each household from a user-specified minimum/maximum number (Figure 2). The age of each child is also randomly selected. Because the children have to go to school and the city is highly networked, the child's public school is randomly assigned, where the locations of the public schools themselves are randomly generated based on the user specified number of such facilities (Figure 2).

For the nuclear family, the total number of E/Rs and the minimum/maximum group size for the E/R communities are user specified (Figure 2a). At least one adult is randomly assigned to interact with each created E/R community. The total number of public schools is selected in the menu (Figure 2a). Figure 1a shows an example of a city consisting of nuclear families and E/R communities. In this case, the E/R community population size ranges from 10 to 20 individuals.

For the extended families, both children and E/Rs are assigned randomly to a household from a user-specified minimum/maximum number (Figure 2b). The assignment is done using the same method as used for the children except the E/Rs are always localized at home. The total number of public schools is also specified in the menu (Figure 2b). Figure 1b shows an example of a city consisting of extended families.

In general, the precise population distribution depends on at least one random variable. The age distributions are uniformly assigned over the specified age groups.

### Construction of the interaction networks

The interaction network between individuals is assigned according to a predefined schedule of each type of individual: adult, youth or E/R.

#### The Adult Networks

**Figure 3:**
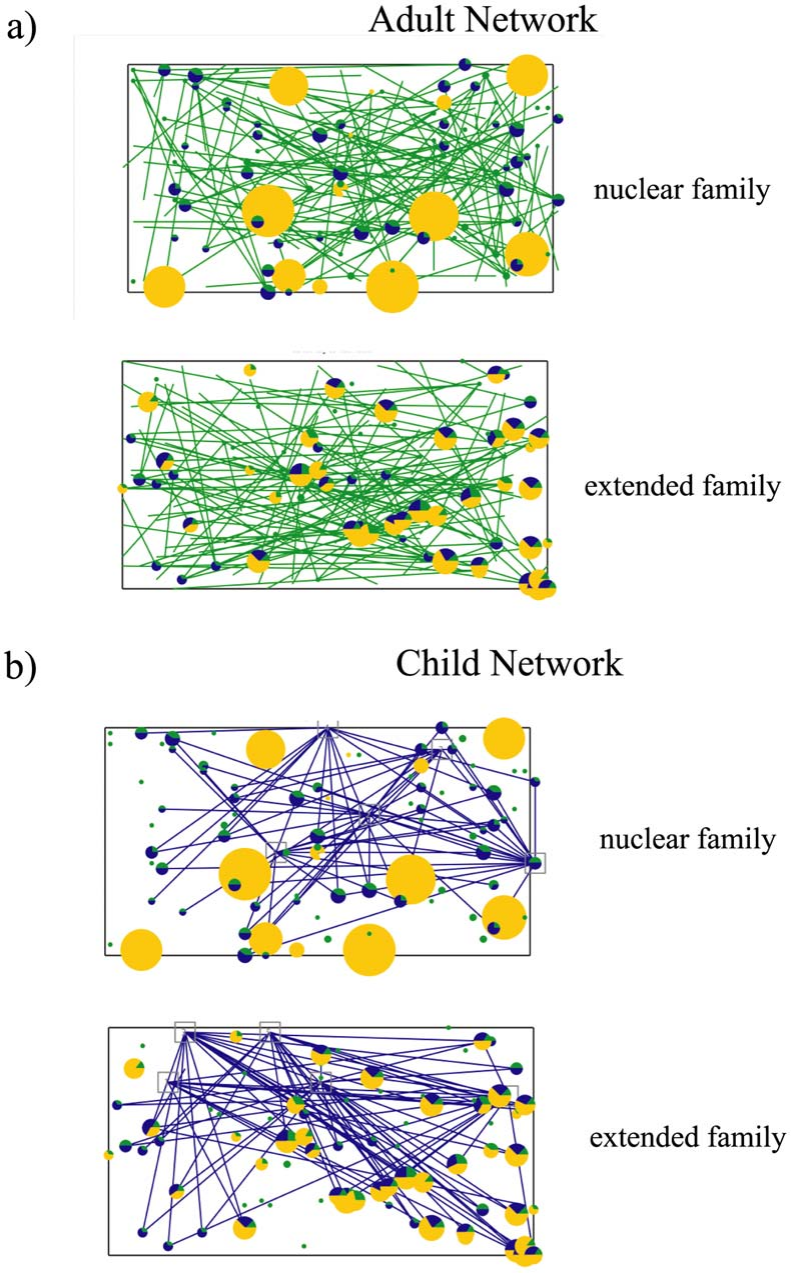
Example of an adult network (a: green lines) and a child network (b: blue lines) in an extended family culture and a nuclear family culture. The adult individuals are indicted by the green dots and their locations at various times of the day are indicated by the green lines that radiate from the initial location of the adult at home. Children are indicated by the blue dots, and the elderly by the yellow dots. The size of the dots indicates the number of people occupying the particular location. The child network includes the institutions (gray squares) where they attend public schools and the connectivity is indicated by the blue lines. The adult and child network connectivity is constructed by the same rules in both the nuclear family and the extended family.

The adult network consists of different locations where and times which the adult spends in a given day. An example of the complete network for the entire group of adults is shown in Figure 3a. The daily activities and network structure for the adults in both societies is generated using the same process. Each adult individual (ages 19 to 55 yrs) is assigned a home, a family and a random number of places that the adult goes during the day. The number of locations that an adult visits from the home and the positions are randomly selected when the city is constructed and the *general whereabouts* (the green lines in Figure 3a) remain the same for all simulations. The *precise whereabouts* are defined later. In essence, we expect that the individuals will move around but they will tend to have a general pattern of movement that is determinable. Figure 3a shows the general whereabouts of the adults (the green lines). In the current constructed cities, the number of alternative locations from home was set to a maximum of four.

#### The Child Networks

Children (ages 0 to 18 yrs) are assumed to be either at home or at school. Hence, for 8 hours of the day, they remain in a public location in close proximity with other school children and for 16 hrs they are located at home (either alone or with family members or neighboring friends). An example of the children's network with public schools is shown in Figure 3b. The network structure (the blue lines in Figure 3b) and connectivity for the children in both types of societies is generated using the same process.

The default conditions assume that the children attend public schools. However; just before running a simulation, the user is queried with an option to choose home schooling. If the “home schooling” option is selected, the public school network is completely cut and the children only interact with other entities within the radius of the home region, presumably immediate family or neighboring friends. Hence, this assumes that the children are educated at home or with nearby families.

#### The E/R Networks

The E/R population (ages 55+) is assumed to be located only at home. It is presumed that they are more sedentary and therefore tend to move in much smaller circles similar to the children. It would be possible to give some of them a more diverse schedule too, but currently, we make this assumption.

### Infection, propagation and disease cycle

The model for the disease consists of three parts: the infection mechanism, the latency period and the decision of fate for an infected individual.

#### Infection Mechanism

The infection mechanism of the model assumes that transfer of the pathogen is via local human contact, be that air-borne or physical contact. As a result, we assume infection only occurs if the proximity between the infected individual (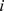) and a potential host (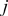 (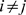)) is sufficiently close that their mutual interaction satisfies the following function
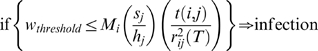
(1)where 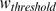 is a weight reflecting the threshold whereupon infection is possible, 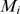 is the *reproduction weight* of the infected individual 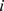, 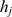 indicates the relative hygiene of the individual 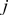 (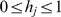; 0 = poor hygiene, 1 = excellent), 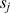 indicates the relative infection susceptibility of individual 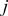 (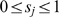; 0 = immune, 1 = guaranteed infection), 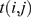 is the joint interaction time between the two individuals (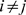), 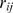 is the mutual distance between the two different individuals and *T* is the *scale of activity* or “temperature” of the city. These parameters will be discussed in more detail later in this section.

#### Latency Period

After infection, a user determined fixed latency period (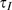) is used to decide how long the infectious agent *i* remains active before the symptoms become apparent while possibly propagating the infection to other individuals. The latency period is queried just before the simulation is begun.

Fate of the infected individual: After a latency period (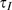), the default conditions for the fate of the infected individual are decided as follows
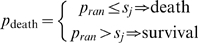
(2)where 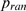 is a random generated number between 0 and 1. Note that when 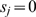, the individual is immune to further infection. In these simulations, we assume that the susceptibility to disease (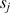) is also indicative of the susceptibility to mortality; which need not be so. In essence, we expect that the hygiene of most individuals after becoming sick is similar enough that the survival at that point becomes a function of how susceptible the individual was before infection. We could also have created a set of independent parameters for the mortality or scaled 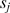 by some user specified weight. Presently, to overcome such issues, the user can select a fixed fate for the death toll (0 to 1) for all individuals where ‘0’ means that all infected recover and ‘1’ means that all infected perish. In this work, we report results associated with Equation 2. Regardless of which model is invoked, whether the victim is immune or dead, the victim is no longer contributing to the spread of the infection; hence, all fatalities can be scaled to reflect a different likelihood of mortality decoupled from the susceptibility if necessary.

We now describe the parameters in Equation (1).

The *precise whereabouts* of an individual is incorporated into the *separation distance* parameter (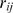). The *separation distance* expresses the distance between the infected agent 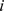 and potential host 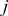 at a given time step corresponding to some specified duration (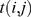) during a given day. For each individual, a vector 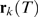 (*k* = *i* or *j*) is constructed at each time step *t*. The vector 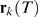 is the sum of two vectors. The first vector defines a fixed distance 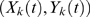; the *general whereabouts* of individual *k* at time *t* in a given day. These fixed vectors correspond to the specific lines that radiate from a given individual in Figure 3. The second vector is a small randomly generated vector 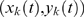 of maximum radius 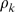. Currently, 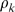 is a fixed value of 0.2 units for all individuals and is increased to 1 unit for all adults who are outside the home. The second vector is weighted by a scalar 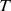 such that 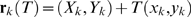. The value of 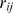 is therefore
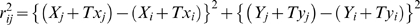
(3)where the time *t* and the temperature *T* are implicit in 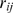. Hence, a person who moves to point 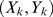 but sits in essentially one place (average 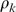 is small) only experiences a slight increase in the range of activity with increasing *T*. However, for that same person, if the average 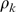 is large at 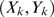, then the range is increased considerably with increasing weight *T*.

The *interaction time* (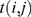) between agent *i* and host *j* is based on the assumption that the possibility of exposure to infection is increased in direct proportion to the amount of time that agent *i* spends in the proximity of host *j* [19] and in inverse proportion to the square of the distance between them; *i.e.*, 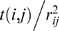. The inverse-square law is a common property for a point source in physical problems such as measuring light intensity. The transmission (or “coupling”) should be strongest for people in close proximity of an infectious agent (in the case of a contact dependent infection). Since infection is also more likely to occur with increased exposure to an infected individual, Equation 1 is weighted by the duration of the interaction time between the two parties.

The *susceptibility factor* (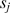) consists of two contributions: a base constant susceptibility for the particular age group (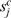) and a variable contribution (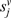) that depends on the individual. Therefore, the range of 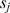 varies from person to person such that 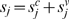, The value for 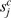 and the range of 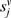 is specified by the user when the city is built and can be modified to different values (Fig. 2c) without changing the configuration of the city.

The *threshold constant* (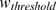) is associated with the hygiene factor (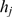) and is made an independent parameter in order to weight the hygiene in such a way as to match a desired pattern of infection and susceptibility. Similar to 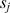; 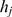 has a constant part and a variable part such that 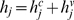. Better hygiene (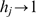), even washing ones hands with soap and clean water [9], [20], should reduce the likelihood of infection, though the particular “hygiene” could vary for different diseases. Since individuals have different susceptibility to a disease, the two parameters are decoupled. It is likely that a specific agent that knows he/she has high susceptibility to a disease (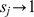) will practice better hygiene; however, the current model makes no assumptions about individual practices and keeps these parameters completely decoupled.

The *reproduction weight* (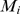) is a measure of the transferability of the disease from the infected individual to other humans. Here the model allows for the fact that some individuals may have habits and/or behaviors that are more effective in transmitting a given disease [11]. Similar to 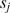 and 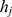, 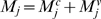. This weight differs from the *reproduction number* (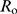) [12], [13], [17], [18], which expresses the mean number of cases generated by an infectious individual. Here, 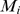 serves only as a weight [18]. For example, if there is no significant infection mechanism between other humans, such as is the current situation with H5N1 avian flu amongst humans, then no infection can occur and the reproduction weight is *M_j_*∼0. The disease will begin and stop with the infected individual. On the other hand; if 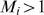, then individual *i* will be 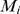 times more likely to infect other individuals. Hence, 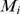 is more of an individual weight [17], though 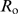 should be a function of the average reproduction weight for all individuals collectively [18], [19].

From Equation 1, prevention through good hygiene (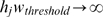) remains the first line of defense. Highly susceptible individuals (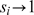) are the most certain to be afflicted but good hygiene and a high threshold can ameliorate the effect. The second line is rapid detection. This depends largely on 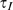, so a disease that operates by stealth is clearly the most dangerous [21] in this model because it increases the overall exposure time and therefore the chance that an infected individual will in turn infect other hosts. If 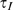 is long and the infection mechanism is highly contagious, then there clearly is little that any policy can do to avoid leaving everything to chance. Likewise, when the threshold (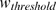) is small, there is no clear hygiene other than complete isolation that will prevent infection.

### Conditions used in this particular study

This study is intended to focus on aging populations, where a large proportion of elderly and small number of young is projected to occur in many developed countries [4]–[8]. Therefore, we have used a very disproportionate E/R population with a small population of youth and we only focus on one such distribution; *L*·100 adults, *L*·80 children and *L*·150 E/Rs, where *L*(>1) is an integer. The behaviors we observed were quite general and, though there are some group dependent effects, reasonable choices for the population distribution did not drastically alter any of the major conclusions made in this work. Therefore, we see no major loss of generality in selecting these parameters.

Because the number of children in each family is randomly generated (Fig. 2ab), a particular population distribution must be found by trial and error. For extended families, the E/Rs are also specified in this way (Fig. 2b) and require multiple trials before a city of the proper proportions is generated. As a result, the populations of all groups were set to their nominal values to within 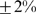 in both family structure scenarios.

Identical configurations were tested both with and without home schooling. By choosing the same layout for both cases, we can rule out any possible layout bias.

The disease profiles are also fixed in this study. The particular parameters used in this study are listed in Table 1 and 2 and their averages are listed in Table 3. The disease profile strongly influences the likelihood of infection and survival. Nevertheless, the threshold constant (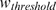) is selectable at the beginning of the simulation and can be adjusted to yield a more or less severe infection cycle by trial and error; depending on the interests of the user.

**Table 1:** The parameters used for the extended families.

Parameter	Variable type	Children	Adults	Elderly	Totals
Population		719	900	1357	2976
Susceptibility	Fixed	0.70	0.50	0.70	
	Variable	0.30	0.50	0.30	
Transmission	Fixed	1.50	1.00	1.50	
	Variable	0.90	1.50	0.90	
Hygiene	Fixed	0.50	0.70	0.50	
	Variable	0.30	0.30	0.30	

Fixed parameters involve a constant term that is independent of the individual. The variable parameters reflect the range of variation within that group. The average values for the whole group are listed in Table 3.

**Table 2 pone-0007221-t002:** The parameters used for the nuclear families.

Parameter	Variable type	Children	Adults	Elderly	Totals
Population		706	900	1350	2956
Susceptibility	Fixed	0.70	0.50	0.70	
	Variable	0.30	0.50	0.30	
Transmission	Fixed	1.50	1.00	1.50	
	Variable	0.90	1.50	0.90	
Hygiene	Fixed	0.50	0.70	0.50	
	Variable	0.30	0.30	0.30	

Fixed parameters involve a constant term that is independent of the individual. The variable parameters reflect the range of variation within that group. The average values for the whole group are listed in Table 3.

**Table 3:** A summary of the average value for various parameters listed in Eqn (1) that are used in the examples giving in text.

Extended family	Age [yrs]	hygiene	susceptibility	Transmission
total:	48.73	0.71	0.82	1.90
child:	8.99	0.65	0.86	1.97
adult:	35.87	0.84	0.75	1.75
Elderly:	78.31	0.65	0.85	1.96
Nuclear family	Age [yrs]	hygiene	susceptibility	transmission
total:	48.71	0.71	0.82	1.89
child:	9.26	0.66	0.85	1.95
adult:	37.99	0.88	0.77	1.81
elderly:	76.48	0.63	0.83	1.91

These averages are listed in terms of age group as well as general values.

**Figure 4:**
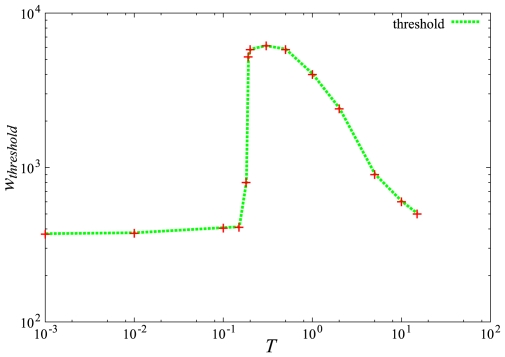
A log-log plot of the threshold (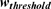) with respect to the temperature for a fixed number of fatalities. There are three temperature regions: *T*<0.2 where the threshold is basically flat, 0.2<*T*<0.5 where the threshold suddenly jumps and reaches a maximum, and *T*>0.5 where it decreases monotonically. The threshold has a very wide range of values near 0.2<*T*<0.4.

Values for 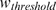 and *T* were selected with an interest in finding critical points where the observed transfer rate could be slowed significantly. Since a small threshold almost guaranteed complete and immediate infection of the entire population, such selections were not considered. The properties of the critical values could be roughly worked out in a log-log plot of 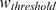 with respect to *T* (see Fig. 4 and Supplement S1).

In this study, the latency period (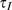) was set to 9 days. For the influenza virus this is twice as long as the typical latency period [17]. Again, the general profiles are reproducible by varying 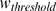 and/or *T* and do not change the general conclusions (see Fig. 4). Since, the specifics of any particular disease cannot be known beforehand, long hidden latency period can be considered the “worst case scenario” for such a pandemic [21].

### Scalability and statistics

The observations tended to be scalable for a given population distribution and parameterization because the construction of the cities always uses the same units of distance in assigning the location of households and individuals. Hence the initial population density is always the same for all constructions and just spreads out evenly with increasing population. The main difference occurs for small *L* where there is far greater noise. The data tended to smooth out with increasing population size.

Furthermore, in general, changing these various profiles (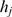, 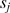, 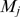, 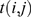, 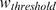) did not appear to change drastically the conclusions because it was always possible to find some 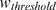 and/or *T* that produced similar infection profiles. For example, see Fig. 4 where all the trials were adjusted to yield the same fatalities (detailed in Supplement S1). The results are also highly convergent, and therefore do not require large samples to see the tendencies.

Therefore, due to this predictability and convergence, we reasoned that we could settle on the profiles listed in Tables 1 through [Table pone-0007221-t002] 3 without any significant loss of generality in the results because the conclusions drawn here largely apply to other profiles. The repeatability of the results suggested that the statistics from a small set of runs would be sufficient to see these tendencies (in this case we only used 10 to 50 trials each, but other tests have used as many as 100 without any unexpected variations). Certainly, although some unusual cases may indeed exist, a sample of the top ten outcomes should, in general, reflect the most commonly expected outcomes.

### Implementation

The program used to implement these model calculations was developed at the International Medical Center of Japan. The program is written in Java and took advantage of the NetBeans development environment (http://www.netbeans.org). An executable version of the program is available on request to researchers upon receiving a signed agreement from the laboratory director to abide by the software license.

## Results

For the nuclear family results, we observe infection results similar to those reported in real case studies [1], [10], [14], [16], [19], [22], [23]. Unlike the nuclear family results, we could not find any similar studies for extended family societies. Many societies in Asia have large populations and a cultural tradition of extended families. However, in the most rapidly developing countries, the migration patterns are in flux [15].

In what follows, we observed the following tendencies.

**Figure 5:**
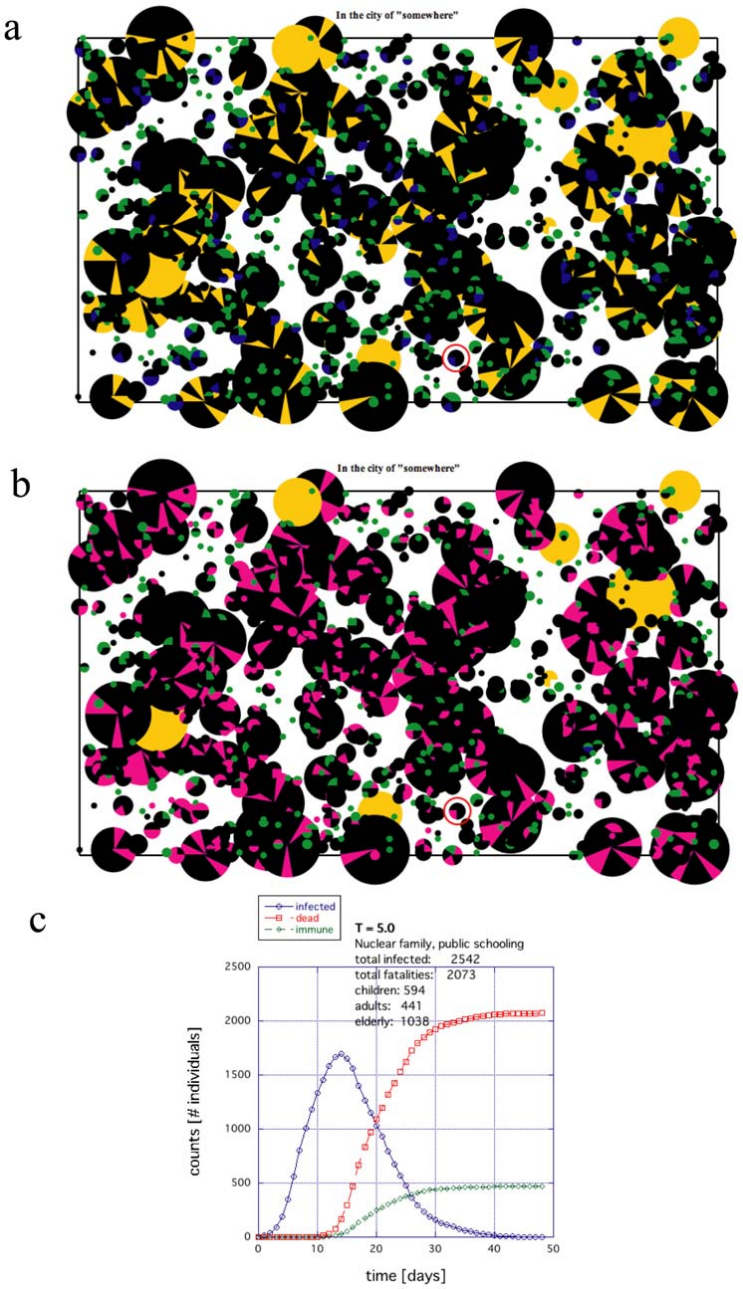
An example of the death toll for the nuclear family society where the E/R are placed together in large facilities and children are sent to public schools. The size of the circles indicates the relative population size. The black indicates those who have died. Yellow indicates E/Rs, blue indicates young, and green indicates adults. (a) Surviving population whether infected or not where black indicates deceased. (b) Population of infected individuals both deceased (black) and recovered (magenta). (c) Time histogram of the rate of infection, death and recovery.

**Figure 6:**
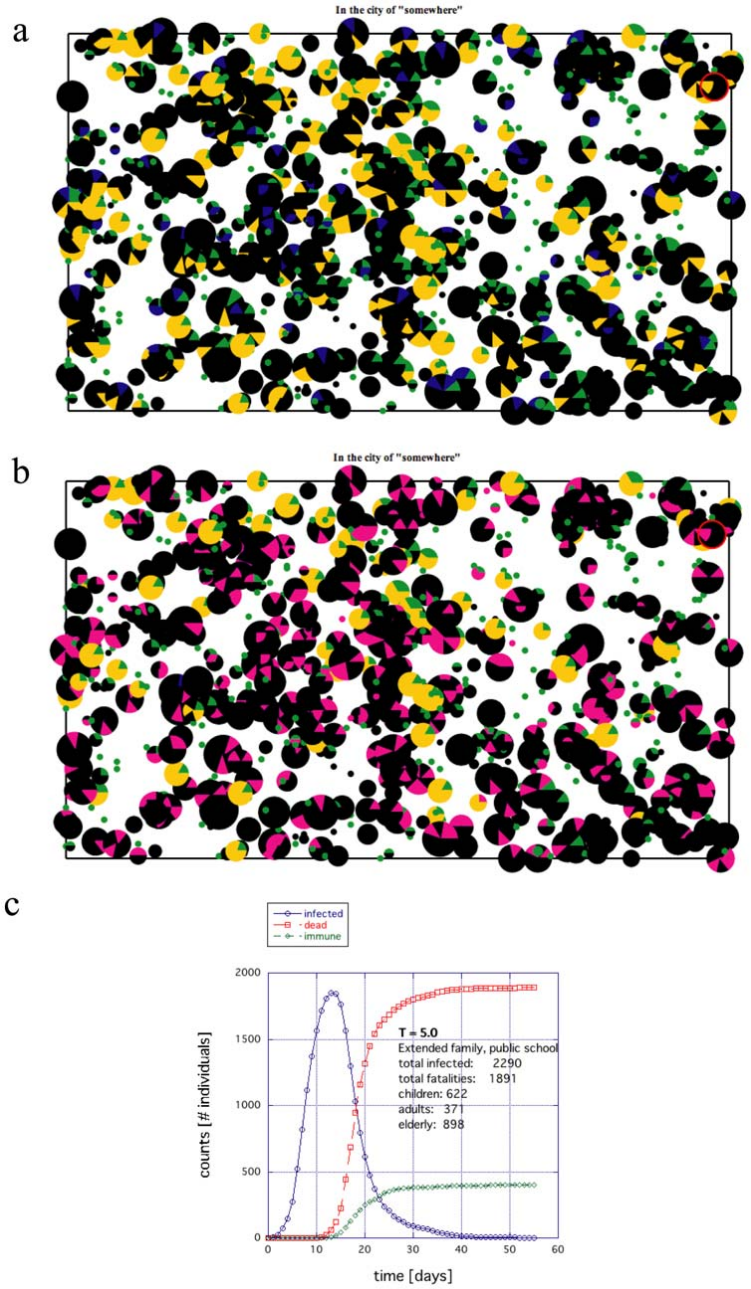
An example of the death toll for an extended family society, where the children go to public schools. In the extended family, the E/Rs are distributed with the families rather than grouping together in small communities. (a) Surviving population whether infected or not where black indicates deceased. (b) Population of infected individuals both deceased (black) and recovered (magenta). (c) Time histogram of the rate of infection, death and recovery.

A significant death toll for the aging population results from placing the elderly and retired in large group facilities. Being especially vulnerable to disease to begin with and highly localized from the start, a substantial death toll of the elderly is almost guaranteed once a disease enters such a facility. An example of this type of die out is shown in Figure 5, where the black sectors reflect deaths (Figs. 5ab), the total infected population (including fatalities) is shown in Fig. 5b and a time histogram of the infection is shown in Fig. 5c. The extended families appeared to do marginally better with children going to large public institutions (Fig. 6), where again the black sectors reflect deaths (Figs. 6ab), the total infected population is shown in Fig. 6b, and a time histogram of the infection is shown in Fig. 6c. The difference between Figs. 5 and 6 is at most only a few percentage points for similar conditions (Supplement S2). When the size of these E/R facilities (nuclear families) was reduced such that the number of E/R individuals was the same as those who lived in the extended families, the fatalities became similar to the extended families and when the maximum size of these E/R facilities was increased from 20 (Fig. 5a) to 100, the consequences were even more devastating (data not shown).

**Figure 7:**
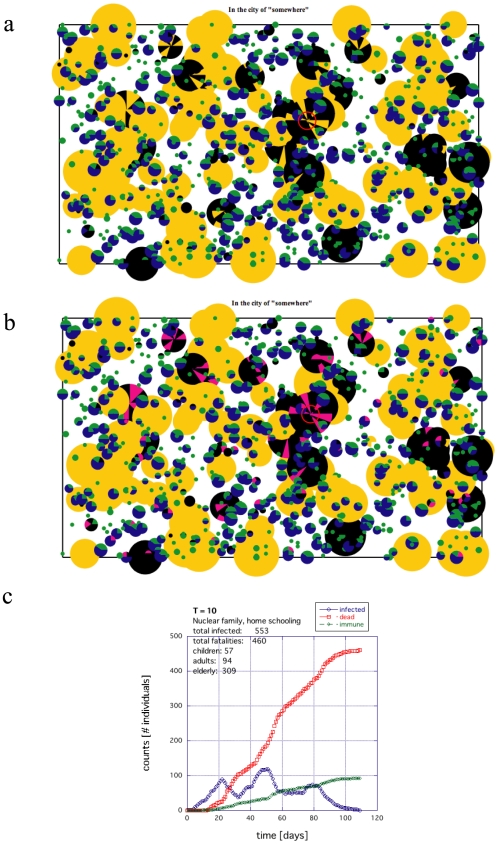
An example of the death toll for the same nuclear family society as in Figure 5 if home schooling is introduced (all other parameters held constant). (a) Surviving population whether infected or not where black indicates deceased. (b) Population of infected individuals both deceased (black) and recovered (magenta). (c) Time histogram of the rate of infection, death and recovery.

**Figure 8:**
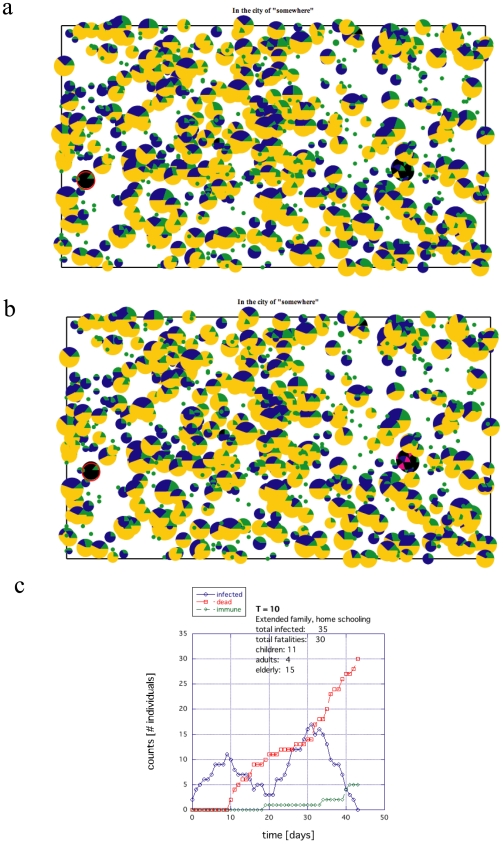
An example of the death toll for the same extended family society as in Figure 6 after home schooling is introduced (all other parameters held constant). (a) Surviving population whether infected or not where black indicates deceased. (b) Population of infected individuals both deceased (black) and recovered (magenta). (c) Time histogram of the rate of infection, death and recovery.

Centralized locations where people interact outside of the household, such as students attending a public school, also drastically affected the results. When the children were limited to home environments, the death toll for the entire population was substantially reduced for both societies (compare Figs. 5 with 7 and Figs. 6 with 8). Moreover, the exposure to infection is reduced significantly. The magenta and black regions are considerably less prominent in Figs. 7b and 8b compared to Figs. 5b and 6b.

The most dramatic impact of home schooling was observed for the extended family societies, where the *maximum* death tolls easily shrank below 1/25^th^ (and typically less than 1/100^th^) that of the public school societies (all other factors held constant): compare Figs. 6 with 8 and Supplement S2. The effect is consistent over temperatures of several orders of magnitude. Nuclear families did significantly better with home schooling, but the *maximum* in fatalities only showed a decrease of 1/2 to 1/3 that of the public school societies (all other factors held constant); compare Figs. 5 with 7. The main reason for this vast difference is that many weak individuals are grouped together in the same place and connected to an adult network that can propagate the disease back and forth between the public schools and the E/R communities. On the other hand, because the extended families could manage their own E/R individuals within the household and educate the children in greater isolation, the extended families could function independently and therefore cut the chain more effectively. Hence, limiting, minimizing or cutting the transmission routes *from all sides* (all age groups) is an important consideration in improving survival.

The results suggest that practices such as home schooling would actually have a significant positive effect on reducing the spread of a disease. This also suggests that permitting flexible work times and allowing the adults to work at home (in jobs where this is possible) would further reduce the extent of the fatalities in a major pandemic.

The death toll strongly depends on the first person infected. If that person does not interact significantly with the population at large, the death toll is likely to be small. On the other hand, a highly active person who comes into sufficiently close contact with many individuals can cause a substantial death toll for any configuration we try. We refer to this as “who's on first” (WF).

In almost all examples, there are cases where isolated individuals become infected and die with no transmission (or limited transmission) of the disease to other members of the population. This happened regardless of such factors as the infectivity, susceptibility, or threshold value of the infectious disease. It entirely depended on the particular shape of the network. Naturally, a very highly infectious agent and a population with a very low threshold were inevitably doomed, but there were still examples even in this set where WF turned out to be isolated enough to prevent any spread. The distributions tended to be in one of two poles; either less than 20 fatalities (about 20% of the cases) or at the maximum (Supplement S2).

For the cases where the selected infected individual turns out to be the most lethal, there are several observations.

First, the profile and character of the infection spread is dependent on the scale of the activity or temperature (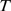), which strongly influences 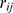 (defined in Equation 1).

**Figure 9:**
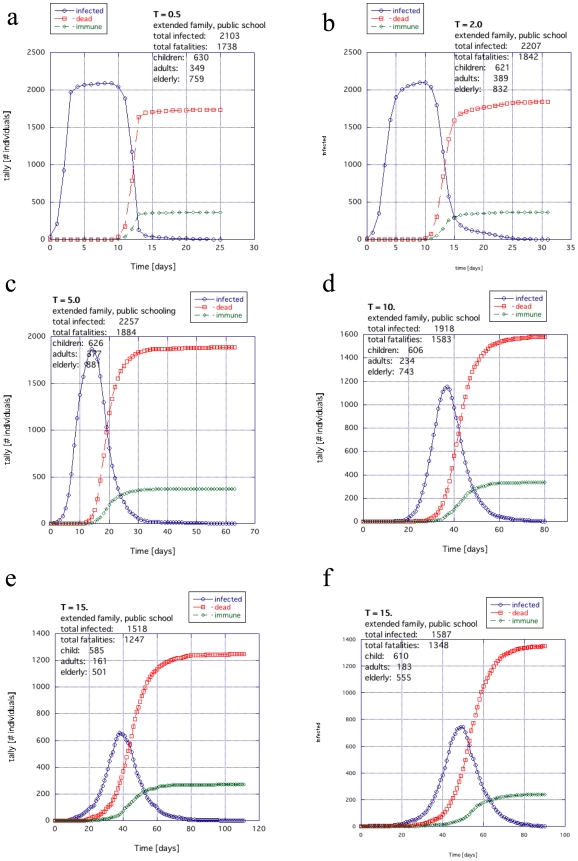
The general trend for temperature with the extended family example when the children go to public institutions: (a) 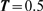, (b) 2.0, (c) 5.0, (d) 10.0 and (e and f) 15.0.

The activity within the city (which we define as “temperature”) has some tendency to diminish the explosive spread and rapid death toll. This is because each agent spends less time interacting with other agents and therefore has less chance to have sufficient contact (reduced coupling). However, a high activity also has the tendency to delocalize the infection, to insure the spread and to prolong the duration of the infection cycle. Nevertheless, because of the slower propagation rates, there are also greater possibilities for intervention. The infection profiles for different temperatures for extended families, where children are sent to public schools, are shown in Figure 9 (details in Supplement S2).

In Figure 9(a–c), the scale of activity is small: (a) *T* = 0.5, (b) 2.0 and (c) 5.0. For the case where the unlucky WF also can spread this infection rapidly through the population, the spread of infection and death tolls are similar; for some cases taking out 1/2 to 2/3s of the population.

When the scale of activity increases to *T* = 10 or *T* = 15, the duration of infection begins to spread out in Figures 9d and 9ef respectively, reflecting the lower degree of sufficient contact. The death toll also drops visibly and there is a much higher likelihood of multiple peaks. This was consistently seen in numerous examples and conditions we tried well beyond those shown in Supplement S2.

**Figure 10 pone-0007221-g010:**
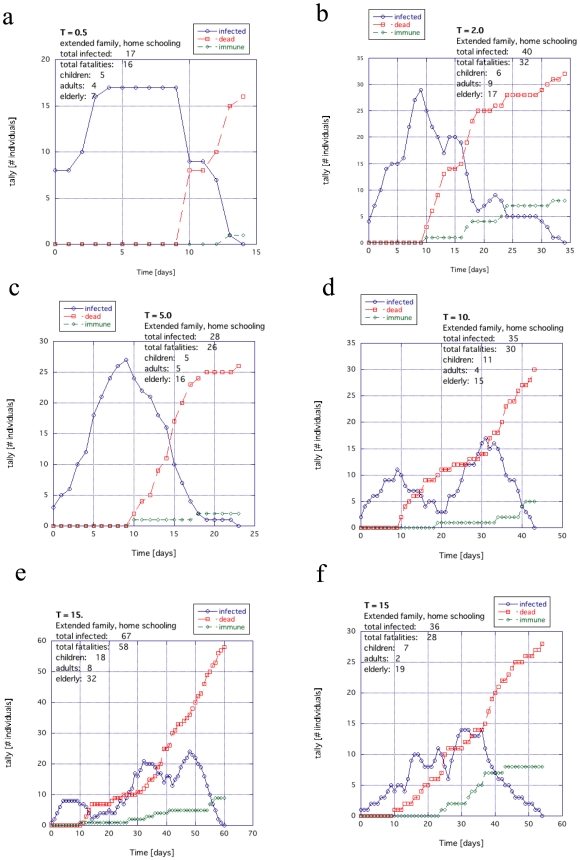
The general trend for temperature for the extended family example when the children are educated at home: (a) 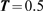, (b) 2.0, (c) 5.0, (d) 10.0 and (e and f) 15.0.

The case where home schooling is applied is shown in Figure 10. The multiple peaks observed in the Figures (blue line) are typical of networks when they are approaching the very edge of functioning. Overall, in the extended family examples, the death tolls were almost completely independent of temperature *T* (Supplement S2).

**Figure 11 pone-0007221-g011:**
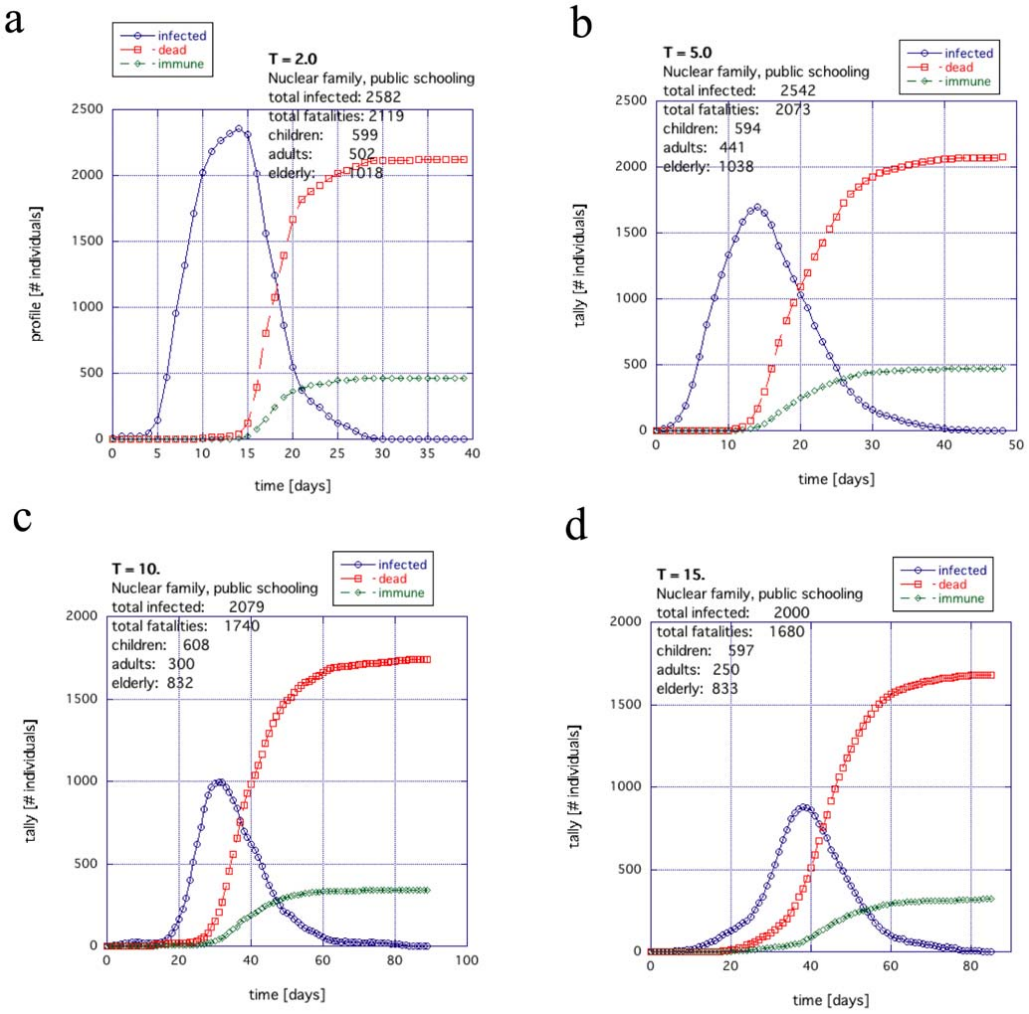
General trend of a nuclear family society when the children are home schooled as a function of temperature; (a) 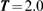, (b) 5.0, (c) 10.0, (d) 15.0.

Figure 11 shows the nuclear family with the children going to public schools. A trend similar to Fig. 9 is also observed where the death toll and corresponding infection are reduced as the temperature is increased. The death toll and infection begin to drop off significantly when *T*>5 (Fig. 11; details in Supplement S2).

**Figure 12:**
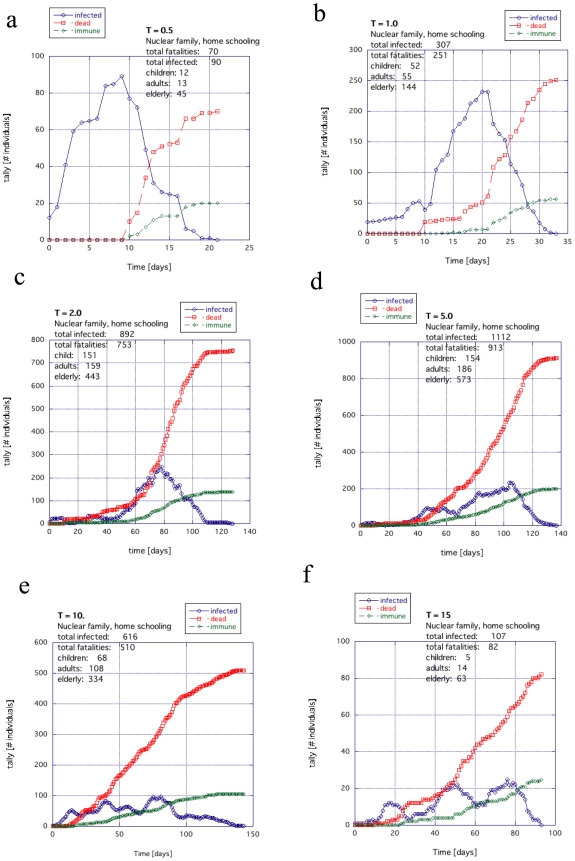
The general trend for temperature for the extended family example when the children are educated at home: (a) 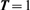, (b) 2.0, (c) 5.0, (d) 10.0 and (e and f) 15.0.

In Fig. 12, the nuclear family with home schooling is shown. Because the networks are at the edge of functional, the profiles tend to have a lot more structure than seen when including public schooling. As the temperature is increased, the infection cycle lengthens and the complexity of this structure becomes more visible. The oscillations for *T* = 10 (Fig. 12d) and 15 (Fig. 12e) are caused by the large communities of E/R becoming infected. Much larger communities can produces several large clean oscillations like this for a well selected *T* and 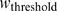. As the temperature increases, the fatalities decrease. Although there was almost no dependence on *T* for the extended family with home schooling (Fig. 10), there is a visible falling off as *T* increases for the nuclear family with home schooling in Fig. 12.

Therefore, Figures 9 through [Fig pone-0007221-g010] [Fig pone-0007221-g011] 12 all suggest that, in terms of temperature dependence, the activity in a city does not necessarily make the situation worse. The infection duration tends to spread out but it appears that this can actually reduce the overall fatalities and infections because there is less interaction time between individuals and their likelihood of coming within significant proximity of each other, for sufficient duration, decrease. This is expected from Equation 1 where the threshold for infection is crossed when proximity is close and interaction time long. With an interaction time that is generally smaller and an interaction distance that is larger on average, the exchange coupling of infection reaches a critical drop off. There is, however, always the same general dependence on WF.

The long latency period (9 days) increases the severity because the “vectors” (infected individuals) have numerous opportunities to infect the same hosts before the symptoms are detected. In this model, the transmission appeared to be primarily dependent on the ability to infect people in immediate proximity; typically the household. Hence, the range between *T* = 1 to *T* = 5 can yield massive fatality for an ill-fated choice of WF in the same infection scenario, city, family structure and population distribution.

Essentially motionless or extremely active societies appeared to produce roughly similar results; though the highly active cities showed a longer duration in the infection time-histogram. This can also be inferred from the threshold plot in Figure 4 where the extreme ends have roughly the same 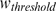. The infection drops for small *T* because the individuals are too far away on average and move around too little to have sufficient coupling. By staying almost still, they effectively “isolate” themselves.

In all the studies, the threshold for resistance to the disease (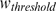 in Equation 1) strongly influenced the way the disease propagated. In the models tested, this parameter was selected to yield complex structure in infection profiles when T>10. The results suggest that hygiene is a major first defense to any infection. Even washing ones hands should have positive effects though with limits [9], [20]. The specific behavior of the infection cycle depended on the particular population and the disease profile. Nevertheless, the larger the value of 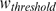, the more it tended to limit the spread of the infection. When values of 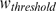 and *T* crossed a critical value, the fatalities dropped off dramatically.

## Discussion

In this work, we studied the difference in survival during a pandemic between an extended family society and a nuclear family society both with the same proportions of aging population and disease susceptibility (Figs. 5, 6, 9 and 11). Such disproportionate aging populations are anticipated in the future in a number of developing and developed societies [4]–[8]. We also considered the effects of localizing children entirely around the home with all other factors were left the same (Figs. 7, 8, 10, 12). The modeled disease was based on the assumed coupling behavior of an air-borne (or contact based) pathogen.

This study indicates some important policy issues to consider.

First, localizing the elderly and retired population in large facilities appears to substantially increase the fatalities in the E/R population. The larger these group facilities were, the more devastating they turned out to be. In developed countries, this type of facility has become rather common. Comparing Figs. 5 with 7 and 6 with 8, this study suggests that it would be better for E/Rs to live in small groups or extended families; at least if other ameliorating factors such as access to vaccination, professional patient care, etc. are not considered.

Second, the infection profile did not appear to have a major impact on the spread of the disease. A far more critical factor appears to be the threshold barrier to infection and the corresponding hygiene of the individuals relative to that. Increasing the hygiene is likely to reduce the ability of a disease to propagate. A more insidious parameter was the incubation time, a parameter that the user could select at the start of a simulation. It became easier to infect a population with long incubation times. Societies with extended families and home schooling appeared to stand a better chance against the worst case scenarios.

Third, as suggested by the substantial decrease in fatalities (Fig. 8a) and infection (Fig. 8b), home schooling offers some benefits. In Fig. 8, the E/R population in each family is quite large. In such a situation, it would be of practical utility to encourage the E/Rs in extended families to participate in educating and caring for the young. The situation where the children attend many large public schools from anywhere in the city offers an excellent breeding ground for spreading an EID. The recent practice of shutting down the public schools during an epidemic is certainly affirmed here. If the infection starts during a holiday break, similar effects as the home schooling are likely to be observed. Likewise, permitting some adults to work at home, rather than commuting [1], should offer further advantages in helping isolate both the young and the old from potential infection. It would suggest that there are some positive effects of home schooling and there are likely to be positive effects for policies that encourage extended families and active participation of the elderly and retired in educating and training the young.

Warehousing in general, though often very efficient and economical, appears to play heavily into the hands of highly asymmetrical odds [24]. Along similar lines, for those who have learned from a hard lesson about backing up data, a distributed approach offers many advantages for insuring the protection of important archives. Hence, distributed approaches have some clear advantages at “preservation” compared to highly centralized approaches.

Since modern societies crucially depend on complex social networks, many of these networks may be difficult or impossible to change. However, neither need we find ways to encourage and promote their further entrenchment.

Comparing the black dots in Fig. 8a (deaths) and magenta dots in Fig. 8b (total infected population) – results for the extended family with home schooling – with the other results in Figs. 5 and 7, it is pertinent to ask why Fig. 8 should turn out to be the best result. We think a reasonable explanation lies in the fact that societies have existed in much smaller groups for the majority of our human evolution [15], [25]. The existence of large cities began within the last 10,000 years [26]–[32] whereas small tribes (or hunter gatherers) have existed within human populations for possibly as long as 4 million years [25]. Among various factors, immunity and defensive action against disease represent a selective advantage for any population [32]–[35]. Therefore, this long evolutionary period of the human race as small tribal units is likely to have selected optimal features in the immune system that favored survival within such environments.

Home schooling differs from quarantining. In this model, the adult individuals carry out normal activities identical to that of the nuclear families. They can easily come home to infect the entire family. The children are not isolated or confined either. In fact, with a T≥5, they can be moving quite a bit locally with neighborhood friends. Only the connectivity and extent of the networks and the structure of the institutions has changed. When children are cut from the network with each other, the results produce Fig. 6 and 8.

Although Figs. 9 to [Fig pone-0007221-g010] [Fig pone-0007221-g011] 12 suggest that large, highly networked cites are likely to be a cauldron that festers and spreads disease – for which there is little recourse available – a large temperature (or “busyness”) in such a population is not necessarily bad. When the activity is large enough, there is actually less chance of fatalities. It is, however, highly dependent on the nature of the disease. We also assume that the hygiene remains the same regardless of the activity. It is not clear that this is necessarily so. Therefore, any particular city may only survive by chance and this should not be taken to suggest that we need not pay attention to the complex web of social interactions.

The death tolls in these simulations are similar to that of the Black Death where it may have wiped out nearly half of the population in some places in the middle ages [36]. The 1918 pandemic was also severe [37]. This is because we selected a high susceptibility and attenuated it with a high threshold. In Table 3, the average susceptibility is 0.82 suggesting as much as an 80% possibility of an infection. Since the model weights the death toll directly by the susceptibility, the death toll would also be similar. The same infection count with a much lower death toll can be achieved by decreasing the susceptibility (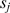) and decreasing the 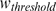. Hence, there are probably many infection scenarios that can appear to yield the same spread yet their actual profiles are quite different.

Finally, we note that the age-related death tolls show that the elderly are preferentially sacrificed in the nuclear family societies. For nuclear families in public school societies, the death tolls are often more than 30% higher than children (Supplement S2). This is because they spend more time in one place and are highly vulnerable. Even for the extended family with public schooling, the death toll for the E/Rs is often similar to that of the children or slightly higher (Supplement S2). In the nuclear family with home schooling, the casualties are much higher for the E/Rs (nearly four fold), though the overall death toll is much lower. This is because the network in the E/R communities remains unchanged with infected adults entering these facilities. In general, the extended family reduces the number of deaths in the E/R population because less of them are grouped together in the same place when a disease enters their abode. These effects can be made far worse by grouping hundreds of E/Rs in single locations. The nuclear family society shows a bias toward greater sacrifice of E/Rs in the event of an infection similar to that modeled here.

We realize that many simplifications have been made in this model. Various forms of congregating behavior such as rock concerts, festivals, political rallies and just “hanging out” were not considered in this model. Ultimately, real scenarios involve a balance between engaging the network and isolation. Nevertheless, the order of magnitude differences seen here suggest that even small improvements are likely to yield a favorable result. Certainly, a far more detailed study with far larger samples and more accurate modeling of the detailed activities would be in order. Nevertheless, because the model is relatively scalable, such extensions are not likely to change the general concepts found here.

### Conclusions

Initially, we would have expected only minor variations. Instead, the results of this study largely affirm that isolating the population into smaller groups or extended family environments offers some degree of overall defense against a pandemic. In a city with rapid transportation, a disease can spread to all parts very rapidly. A substantial reduction in infection and mortality is observed when both the elderly/retired and children are cut off from the highly connected network of such cities. It suggests that localizing the elderly and retired in large facilities can produce undesirable side effects when such a broad network is present. It also suggests that positive effects can result from home schooling, particularly for the extended family communities. Since social networks represent a major means of propagation, limiting the congregation of high risk groups such as the old or young and increasing the relative hygiene of the population as a whole appear to significantly reduce the spread of any typical air-borne pathogen.

Another surprise was to learn that a city of busy people is not necessarily bad for survival in a pandemic because the interaction time between individuals is reduced.

It is important to remind the reader that this is a theoretical study intended to model the generic features of given diseases and the general features of various social environments. The complexities of the real world often thwart even the best theoretical models. A deeper consideration of the complexities, further testing and revision of the model, and serious reflection are all warranted.

Nevertheless, some of the results observed in this work were astoundingly dramatic. In the coming years, many baby-boomers are expected to retire and, in general, the elderly and retired population is expected to increase in many developed countries [4], [5]. Whatever limitations may lurk within the current theoretical model and its framework, these observations seem to warrant further reflection in the areas of city planning and in policy making.

## Supporting Information

Supplement S1 The dependence of w_threshold_ with respect to T for fixed average fatalities
(0.12 MB PDF)

Supplement S2 The death toll and infection profiles for extended and nuclear families as a function of activity (T)
(0.11 MB PDF)
