# Supplementary material for: Home Educating in an Extended Family Culture and Aging Society May Fare Best during a Pandemic
Source: PLoS One. 2009 Sep 28;4(9):e7221. doi: 10.1371/journal.pone.0007221 (PMC2745700; doi:10.1371/journal.pone.0007221)
Supplement: Supplement S1 — The dependence of wthreshold with respect to T for fixed average fatalities (0.12 MB PDF) [file pone.0007221.s001.pdf]

S1: Home educating in an extended family culture and aging society may fair best during a pandemic
Supplement 1: The dependence of $w_{threshold}$ with respect to T for fixed average fatalities

Wayne Dawson[1] and Kenji Yamamoto.
International Medical Center of Japan, 1-21-1 Toyama, Shinjuku-ku, Tokyo 162-8655

Notes:
The term “inf max” means the maximum number of individuals infected in a single day, “immune” means the number of individuals that became infected and survived, “inf peak” means the position where “inf max” reached the maximum and duration is the total number of days between the first infection and the time when the number of infections reached zero. The term “schooling” refers to whether the simulation was done with the children going to public school (public) or whether the option for home schooling was selected (home). Here, we only test public schools. Abbreviations under the column peaks indicate the apparent structure of the infection: sq wv (square wave shaped), s (sharp) and w (weak). The number preceding this reference indicates how many such peaks were observed.

For this data, the outcomes that generated few or no die outs (death toll < 20) were discarded. In the five trials for each temperature that are listed, all of them were sampled sequentially and in general produce a rather similar range. The threshold was fit to the total death toll (the column marked in brown).

data	death toll				Inf peak max	immune	inf peak [day #]	Duration [days]	thresh	temp	Schooling	peaks
	children	adults	elderly	total								
0	607	366	689	1662	2005	343	9	20	370	0.001	Public	sq wv
1	592	366	704	1662	2005	343	7	18				sq wv
2	605	345	699	1650	2005	345	7	18				sq wv
3	595	355	730	1680	2023	345	9	21				sq wv
4	621	340	691	1652	2001	355	10	21				sq wv

[1] Current institution: Bio-information Engineering Laboratory, Department of Agricultural and Biotechnology, Graduate School of Agriculture and Life Sciences, The University of Tokyo, Yayoi 1-1-1, Tokyo 113

0	601	332	701	1634	1997	363	9	20	375	0.010	Public	sq wv
1	593	337	732	1662	2012	352	9	21				sq wv
2	606	361	690	1657	1997	340	9	20				sq wv
3	583	361	681	1625	1997	372	8	19				sq wv
4	605	344	704	1653	1997	344	7	18				sq wv
0	610	315	715	1640	1986	369	11	24	405	0.100	Public	sq wv
1	615	348	700	1663	1982	324	9	21				sq wv
2	603	340	715	1658	1987	329	9	20				sq wv
3	596	341	713	1650	1987	337	8	19				sq wv
4	590	344	724	1658	1986	328	8	19				sq wv
0	605	343	710	1658	1989	339	10	23	410	0.150	Public	sq wv
1	600	336	732	1668	1997	357	9	25				sq wv
2	603	354	701	1658	1993	364	9	25				sq wv
3	603	341	698	1642	1966	349	9	24				sq wv
4	594	337	716	1647	1961	349	10	24				sq wv
0	592	347	685	1624	1863	370	11	32	460	0.170	Public	1sw
1	596	335	726	1657	1799	370	12	35				1sw
2	616	351	764	1731	1865	339	11	33				1sw
3	594	351	827	1772	2007	381	10	36				1sw
4	585	322	688	1595	1804	365	10	25				1sw
0	611	335	700	1646	1621	346	16	40	800	0.180	Public	1sw
1	599	332	694	1625	1655	341	14	40				1sw
2	601	345	786	1732	1551	342	15	39				1sw
3	605	342	777	1724	1722	347	20	41				1sw

4	596	360	799	1644	1755	372	23	44				1sw
5	612	343	725	1680	1640	330	14	44				1sw
0	511	308	744	1663	1332	356	24	52	5200	0.190	Public	1sw
1	603	306	755	1664	1288	359	17	51				1sw
2	591	312	757	1660	1345	353	33	73				1sw
3	606	304	734	1644	1288	354	26	64				1sw
4	615	305	678	1598	1241	326	16	53				1sw
0	576	316	726	1618	1385	369	16	41	5800	0.200	Public	1s
1	614	318	715	1647	1347	313	20	48				1s
2	593	312	695	1600	1485	354	15	41				1s
3	607	322	722	1651	1422	309	21	51				1s
4	621	322	668	1611	1302	298	19	48				1s
0	596	355	753	1704	1712	364	14	52	6100	0.300	Public	1s
1	600	357	681	1638	1651	337	12	40				1s
2	597	378	818	1793	1845	353	11	40				1s
3	607	358	741	1706	1738	358	18	47				1s
4	581	376	716	1673	1723	384	11	32				1s
0	596	359	690	1645	1638	334	13	42	5800	0.500	Public	1s
1	594	347	692	1633	1715	363	16	42				1s
2	582	345	670	1597	1629	362	14	40				1s
3	599	324	632	1555	1567	314	12	36				1s
4	604	331	675	1610	1677	321	11	35				1s
0	603	369	690	1662	1627	326	12	53	4000	1.000	Public	1s
1	591	393	777	1761	1686	355	12	41				1s

2	603	381	728	1712	1608	359	16	48				1s
3	591	385	725	1701	1661	363	12	35				1s
4	598	338	695	1631	1689	353	13	37				1s
0	602	322	727	1651	1380	360	15	43	2400	2.000	Public	1s
1	593	336	695	1624	1389	361	13	43				1s
2	603	381	728	1712	1608	359	16	48				1s
3	593	326	669	1588	1388	338	25	50				1s
4	615	326	593	1534	1408	315	26	50				1s
0	613	297	690	1600	1232	331	16	48	900	5.000	Public	1s
1	581	284	649	1514	1112	352	28	65				1s
2	585	307	752	1644	1255	385	33	75				1s
3	607	311	785	1703	1214	355	16	59				1s
4	589	314	807	1710	1304	354	17	59				1s
0	581	251	758	1590	919	341	33	83	600	10.000	Public	1s
1	605	253	733	1591	1004	306	23	70				1s
2	604	259	604	1687	969	340	50	98				1s
3	597	268	751	1616	926	327	24	70				1s
4	602	271	714	1587	975	303	46	115				1s
1	587	263	847	1697	769	346	50	101	500	15.000	Public	1w
3	597	250	833	1680	877	320	38	86				1w
4	589	236	817	1642	862	356	40	101				1w
7	583	266	889	1738	724	333	27	100				1w
4	589	236	817	1642	862	356	40	101				1w
9	604	248	795	1647	871	307	36	96				1w

Table S1-1. A determination of the dependence of $w_{threshold}$ with respect to the temperature (T) for various values.

temp [au]	thresh [au]
0.001	370
0.010	375
0.100	405
0.150	410
0.180	800
0.190	5200
0.200	5800
0.300	6100
0.500	5800
1.000	4000
2.000	2400
5.000	900
10.000	600
15.000	500

Table S1-2. A summary of $w_{threshold}$ vs T from Table S1-1.

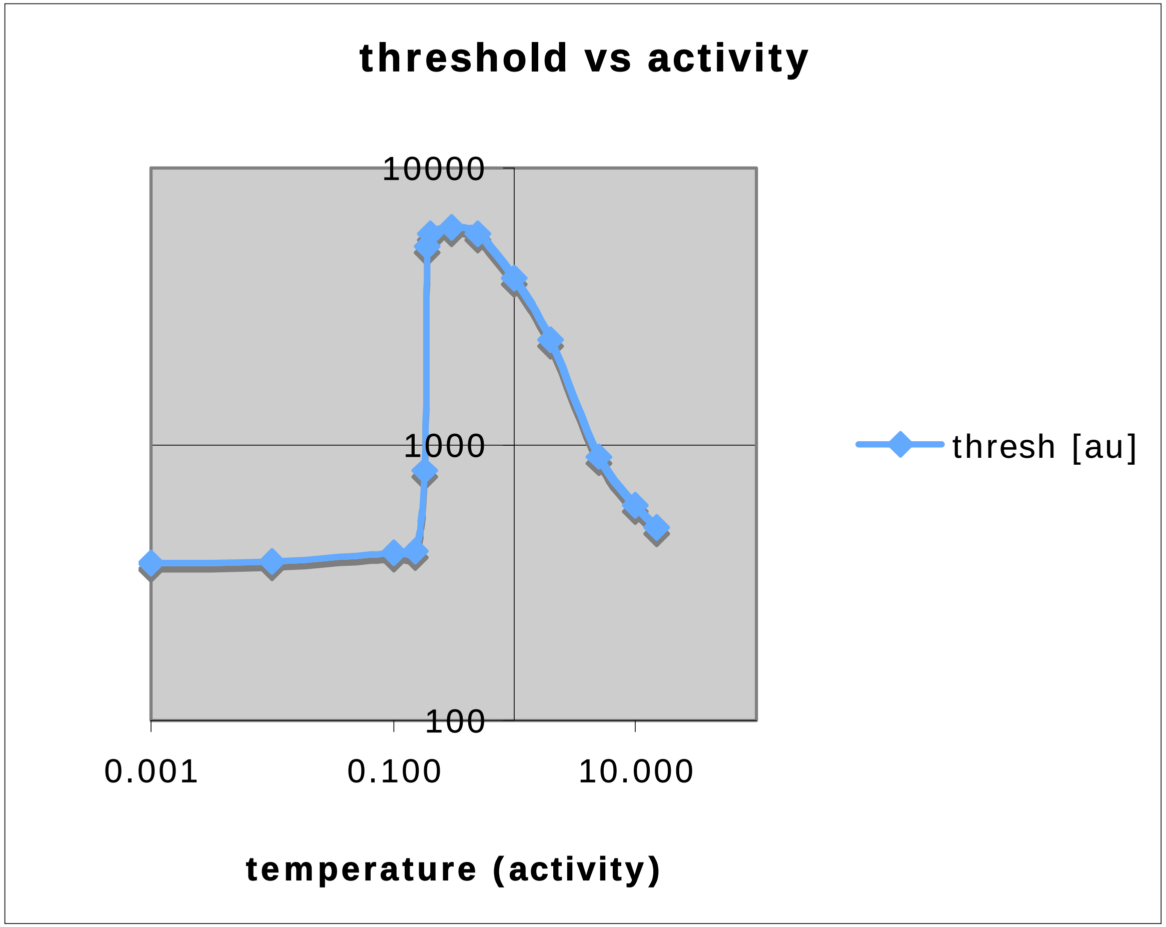

Figure S1-1. A plot of the data in Table S1-2 (same as Figure 4 main manuscript).
