# Supplementary material for: Home Educating in an Extended Family Culture and Aging Society May Fare Best during a Pandemic
Source: PLoS One. 2009 Sep 28;4(9):e7221. doi: 10.1371/journal.pone.0007221 (PMC2745700; doi:10.1371/journal.pone.0007221)
Supplement: Supplement S2 — The death toll and infection profiles for extended and nuclear families as a function of activity (T) (0.11 MB PDF) [file pone.0007221.s002.pdf]

# S2: Home educating in an extended family culture and aging society may fair best during a pandemic
## Supplement 2: The death toll and infection profiles for extended and nuclear families as a function of activity (*T*)

Wayne Dawson[1] and Kenji Yamamoto.
International Medical Center of Japan, 1-21-1 Toyama, Shinjuku-ku, Tokyo 162-8655

Notes:
The term "inf max" means the maximum number of individuals infected in a single day, "immune" means the number of individuals that became infected and survived, "inf peak" means the position where "inf max" was the maximum and duration is the total number of days between the first infection and the time when the number of infections reached zero. The term "schooling" refers to whether the simulation was done with the children going to public school (Public) or whether the option for home schooling was selected (Home).

Data is presented in the way it was taken in the runs. In general, it can be seen that there are basically two ranges for the data: one set shows the number of fatalities was either less than 30 and the other shows a very large number of fatalities. Of course for home schooling with the extended families, there were only quantities of less than or equal to 30 in these trials. It is reasonable to think that there might just so happen to be some who's on first where the connectivity causes some chain reaction and wipes out many extended family units. However, the number of these types of hits is certainly small and compared to the frequency in which massive die outs are observed with the nuclear family, it is still clearly an advantage to use this strategy to maximize the probability of survival for the largest number of possible configurations.

Abbreviations under the column peaks indicate the apparent structure of the infection: square (square wave shaped), s (sharp), w (weak), d (diffuse), "lt" (long tail) and "--" (undefined). The number that precedes the letter indicates the number of such peaks. The order also indicates the right to left appearance of the peaks.

---

[1] Current address: Bio-information Engineering Laboratory, Department of Agricultural and Biotechnology, Graduate School of Agriculture and Life Sciences, The University of Tokyo, Yayoi 1-1-1, Tokyo 113

## Extended families

| Data | death toll children | adults | elderly | Total | inf max | immune | inf peak | duration | thresh | temp | Schooling | peaks |
|---|---|---|---|---|---|---|---|---|---|---|---|---|
| 0 | 0 | 0 | 3 | 3 | 4 | 1 | 0 | 11 | 500 | 0.1 | Public | -- |
| 1 | 605 | 99 | 696 | 1400 | 1654 | 245 | 8 | 19 | | | | square |
| 2 | 613 | 95 | 702 | 1410 | 1653 | 244 | 7 | 21 | | | | square |
| 3 | 599 | 89 | 697 | 1385 | 1385 | 269 | 7 | 18 | | | | square |
| 4 | 609 | 98 | 706 | 1413 | 1658 | 245 | 8 | 19 | | | | square |
| 5 | 0 | 0 | 3 | 3 | 4 | 1 | 0 | 11 | | | | -- |
| 6 | 589 | 93 | 699 | 1381 | 1654 | 273 | 8 | 19 | | | | square |
| 7 | 574 | 93 | 706 | 1373 | 1654 | 281 | 8 | 19 | | | | square |
| 8 | 0 | 0 | 0 | 1 | 1 | 1 | 0 | 11 | | | | -- |
| 9 | 581 | 94 | 689 | 1364 | 1654 | 290 | 7 | 18 | | | | square |
| 10 | 586 | 88 | 707 | 1381 | 1654 | 273 | 7 | 18 | | | | square |

Table S2-1. Extended family with *T*=0.1 and children attend the public schools.

| Data | death toll children | adults | elderly | Total | inf max | immune | inf peak | duration | thresh | temp | Schooling | peaks |
|---|---|---|---|---|---|---|---|---|---|---|---|---|
| 0 | 0 | 1 | 5 | 6 | 6 | 0 | 0 | 11 | 500 | 0.5 | Public | -- |
| 1 | 601 | 338 | 787 | 1726 | 2105 | 389 | 9 | 22 | | | | sq wv |
| 2 | 612 | 374 | 776 | 1762 | 2096 | 340 | 8 | 23 | | | | sq wv |
| 3 | 630 | 349 | 759 | 1738 | 2090 | 365 | 9 | 26 | | | | sq wv |
| 4 | 0 | 1 | 0 | 1 | 1 | 0 | 0 | 11 | | | | -- |
| 5 | 632 | 363 | 773 | 1768 | 2084 | 337 | 9 | 25 | | | | sq wv |
| 6 | 601 | 359 | 767 | 1727 | 2095 | 382 | 9 | 28 | | | | sq wv |
| 7 | 619 | 351 | 747 | 1717 | 2095 | 381 | 9 | 22 | | | | sq wv |
| 8 | 634 | 352 | 771 | 1757 | 2105 | 358 | 9 | 24 | | | | sq wv |
| 9 | 612 | 364 | 767 | 1743 | 2094 | 364 | 9 | 27 | | | | sq wv |
| 10 | 618 | 361 | 765 | 1744 | 2091 | 358 | 8 | 24 | | | | sq wv |

Table S2-2. Extended family with $T$=0.5 and children attend the public schools.

| Data | death toll children | adults | elderly | Total | inf max | immune | inf peak | duration | thresh | temp | Schooling | peaks |
|---|---|---|---|---|---|---|---|---|---|---|---|---|
| 0 | 0 | 0 | 3 | 3 | 4 | 1 | 0 | 11 | 500 | 2 | Public | -- |
| 1 | 629 | 398 | 827 | 1854 | 2128 | 380 | 15 | 34 | | | | 1s |
| 2 | 614 | 377 | 814 | 1805 | 2096 | 364 | 10 | 27 | | | | 1s |
| 3 | 614 | 407 | 836 | 1857 | 2128 | 378 | 11 | 29 | | | | 1s |
| 4 | 621 | 389 | 832 | 1842 | 2100 | 365 | 10 | 32 | | | | 1s |
| 5 | 632 | 399 | 785 | 1816 | 2102 | 385 | 10 | 38 | | | | 1s |
| 6 | 613 | 398 | 808 | 1819 | 2135 | 394 | 11 | 30 | | | | 1s |
| 7 | 620 | 389 | 842 | 1851 | 2104 | 378 | 10 | 35 | | | | 1s |
| 8 | 622 | 382 | 806 | 1810 | 2087 | 390 | 9 | 34 | | | | 1s |
| 9 | 613 | 389 | 843 | 1845 | 2106 | 385 | 10 | 36 | | | | 1s |
| 10 | 628 | 381 | 797 | 1806 | 2108 | 391 | 9 | 28 | | | | 1s |

Table S2-3. Extended family with *T*=2 and children attend the public schools.

| Data | death toll children | adults | elderly | Total | inf max | immune | inf peak | duration | thresh | temp | Schooling | peaks |
|---|---|---|---|---|---|---|---|---|---|---|---|---|
| 0 | 632 | 355 | 870 | 1857 | 1864 | 357 | 14 | 61 | 500 | 5 | Public | 1s |
| 1 | 618 | 374 | 875 | 1867 | 1927 | 415 | 14 | 67 | | | | 1s |
| 2 | 616 | 375 | 891 | 1882 | 1868 | 376 | 14 | 49 | | | | 1s |
| 3 | 623 | 368 | 876 | 1867 | 1909 | 367 | 20 | 48 | | | | 1s |
| 4 | 0 | 1 | 0 | 1 | 1 | 0 | 1 | 11 | | | | -- |
| 5 | 626 | 377 | 881 | 1884 | 1864 | 373 | 14 | 64 | | | | 1s |
| 6 | 600 | 373 | 901 | 1874 | 1850 | 391 | 13 | 54 | | | | 1s |
| 7 | 618 | 355 | 906 | 1879 | 1871 | 375 | 20 | 54 | | | | 1s |
| 8 | 622 | 371 | 898 | 1891 | 1848 | 399 | 13 | 56 | | | | 1s |
| 9 | 0 | 1 | 0 | 1 | 1 | 0 | 1 | 11 | | | | -- |
| 10 | 615 | 371 | 877 | 1863 | 1886 | 386 | 18 | 71 | | | | 1s |

Table S2-4. Extended family with *T*=5 and children attend the public schools.

| Data | death toll children | adults | elderly | Total | inf max | immune | inf peak | duration | thresh | temp | Schooling | peaks |
|---|---|---|---|---|---|---|---|---|---|---|---|---|
| 0 | 606 | 268 | 778 | 1653 | 1136 | 358 | 29 | 75 | 500 | 10 | Public | 1s |
| 1 | 0 | 1 | 0 | 1 | 1 | 0 | 1 | 11 | | | | -- |
| 2 | 0 | 1 | 0 | 1 | 1 | 0 | 1 | 11 | | | | -- |
| 3 | 622 | 238 | 716 | 1576 | 1141 | 304 | 23 | 62 | | | | 1s |
| 4 | 604 | 252 | 749 | 1605 | 1154 | 315 | 44 | 100 | | | | 1s |
| 5 | 605 | 239 | 782 | 1626 | 1216 | 322 | 22 | 77 | | | | 1s |
| 6 | 621 | 282 | 733 | 1636 | 1191 | 324 | 21 | 67 | | | | 1s |
| 7 | 606 | 234 | 743 | 1583 | 1155 | 335 | 37 | 81 | | | | 1s |
| 8 | 629 | 237 | 802 | 1668 | 1150 | 300 | 22 | 76 | | | | 1s |
| 9 | 606 | 241 | 743 | 1590 | 1193 | 346 | 22 | 63 | | | | 1s |
| 10 | 0 | 0 | 0 | 0 | 0 | 1 | 1 | 11 | | | | -- |

Table S2-5. Extended family with *T*=10 and children attend the public schools.

| Data | death toll children | adults | elderly | Total | inf max | immune | inf peak | duration | thresh | temp | Schooling | peaks |
|---|---|---|---|---|---|---|---|---|---|---|---|---|
| 0 | 585 | 161 | 501 | 1247 | 659 | 271 | 38 | 112 | 500 | 15 | Public | 1sd |
| 1 | 606 | 165 | 511 | 1282 | 616 | 255 | 30 | 69 | | | | 1sd |
| 2 | 611 | 170 | 534 | 1315 | 698 | 241 | 41 | 85 | | | | 1sd |
| 3 | 601 | 168 | 518 | 1287 | 701 | 239 | 31 | 77 | | | | 1sd |
| 4 | 0 | 1 | 0 | 1 | 1 | 0 | 1 | 11 | | | | -- |
| 5 | 0 | 1 | 2 | 3 | 3 | 0 | 5 | 16 | | | | -- |
| 6 | 583 | 150 | 558 | 1291 | 677 | 259 | 43 | 87 | | | | 1sd |
| 7 | 0 | 2 | 0 | 2 | 2 | 0 | 7 | 18 | | | | -- |
| 8 | 610 | 183 | 555 | 1348 | 743 | 239 | 49 | 91 | | | | 1sd |
| 9 | 0 | 3 | 4 | 7 | 7 | 1 | 9 | 23 | | | | 1w |
| 10 | 604 | 172 | 533 | 1309 | 712 | 244 | 38 | 115 | | | | 1sd |

Table S2-6. Extended family with *T*=15 and children attend the public schools.

| Data | death toll children | adults | elderly | Total | inf max | immune | inf peak | duration | thresh | temp | Schooling | peaks |
|---|---|---|---|---|---|---|---|---|---|---|---|---|
| 0 | 3 | 0 | 0 | 3 | 3 | 0 | 0 | 11 | 500 | 0.1 | Home | -- |
| 1 | 0 | 1 | 4 | 5 | 7 | 2 | 1 | 12 | | | | -- |
| 2 | 0 | 0 | 0 | 0 | 1 | 1 | 0 | 11 | | | | -- |
| 3 | 0 | 2 | 4 | 6 | 6 | 0 | 0 | 11 | | | | -- |
| 4 | 0 | 1 | 0 | 1 | 1 | 0 | 0 | 11 | | | | -- |
| 5 | 1 | 2 | 2 | 5 | 6 | 1 | 1 | 12 | | | | -- |
| 6 | 0 | 1 | 0 | 1 | 1 | 0 | 0 | 11 | | | | -- |
| 7 | 5 | 1 | 0 | 6 | 6 | 0 | 0 | 11 | | | | -- |
| 8 | 1 | 1 | 0 | 2 | 2 | 0 | 0 | 11 | | | | -- |
| 9 | 0 | 1 | 5 | 6 | 6 | 0 | 0 | 11 | | | | -- |
| 10 | 0 | 1 | 0 | 1 | 1 | 0 | 0 | 11 | | | | -- |
| 11 | 0 | 1 | 3 | 4 | 4 | 0 | 0 | 11 | | | | -- |

Table S2-7. Extended family with *T*=0.1 and children are educated at home.

| Data | death toll | | | Total | inf max | immune | inf peak | duration | thresh | temp | Schooling | peaks |
|---|---|---|---|---|---|---|---|---|---|---|---|---|
| | children | adults | elderly | | | | | | | | | |
| 0 | 1 | 0 | 3 | 4 | 7 | 3 | 1 | 12 | 500 | 0.5 | Home | -- |
| 1 | 5 | 4 | 7 | 16 | 17 | 1 | 4 | 15 | | | | -- |
| 2 | 2 | 1 | 3 | 6 | 9 | 3 | 0 | 11 | | | | -- |
| 3 | 2 | 0 | 3 | 5 | 6 | 1 | 0 | 11 | | | | -- |
| 4 | 0 | 2 | 4 | 6 | 6 | 0 | 1 | 12 | | | | -- |
| 5 | 2 | 1 | 2 | 5 | 8 | 3 | 1 | 12 | | | | -- |
| 6 | 2 | 0 | 4 | 6 | 9 | 3 | 0 | 11 | | | | -- |
| 7 | 0 | 0 | 4 | 4 | 6 | 2 | 1 | 12 | | | | -- |
| 8 | 3 | 2 | 7 | 12 | 15 | 3 | 0 | 11 | | | | -- |
| 9 | 2 | 1 | 4 | 7 | 8 | 1 | 1 | 12 | | | | -- |
| 10 | 0 | 1 | 5 | 6 | 7 | 1 | 0 | 11 | | | | -- |

Table S2-8. Extended family with $T$=0.5 and children are educated at home.

| Data | death toll children | adults | elderly | Total | inf max | immune | inf peak | duration | thresh | temp | Schooling | peaks |
|---|---|---|---|---|---|---|---|---|---|---|---|---|
| 0 | 2 | 5 | 12 | 19 | 17 | 3 | 9 | 27 | 500 | 2 | Home | 1w |
| 1 | 0 | 1 | 2 | 3 | 4 | 1 | 2 | 13 | | | | -- |
| 2 | 6 | 3 | 4 | 13 | 14 | 1 | 2 | 13 | | | | -- |
| 3 | 3 | 1 | 0 | 4 | 5 | 1 | 4 | 15 | | | | -- |
| 4 | 6 | 9 | 17 | 32 | 29 | 8 | 9 | 27 | | | | 1w |
| 5 | 3 | 0 | 0 | 3 | 4 | 1 | 0 | 11 | | | | -- |
| 6 | 2 | 1 | 4 | 7 | 7 | 0 | 1 | 12 | | | | -- |
| 7 | 1 | 3 | 4 | 8 | 8 | 2 | 9 | 22 | | | | -- |
| 8 | 0 | 1 | 5 | 6 | 7 | 1 | 1 | 12 | | | | -- |
| 9 | 2 | 2 | 4 | 8 | 8 | 0 | 2 | 13 | | | | -- |
| 10 | 0 | 1 | 2 | 3 | 5 | 2 | 0 | 11 | | | | -- |
| 11 | 3 | 3 | 6 | 12 | 10 | 4 | 11 | 22 | | | | -- |
| 12 | 1 | 2 | 5 | 8 | 8 | 0 | 1 | 12 | | | | -- |
| 13 | 0 | 0 | 4 | 4 | 5 | 1 | 1 | 13 | | | | -- |
| 14 | 2 | 1 | 0 | 3 | 3 | 0 | 3 | 14 | | | | -- |
| 15 | 0 | 1 | 0 | 1 | 1 | 0 | 0 | 11 | | | | -- |
| 16 | 3 | 1 | 9 | 13 | 14 | 2 | 5 | 26 | | | | -- |
| 17 | 2 | 1 | 4 | 7 | 9 | 2 | 7 | 18 | | | | -- |
| 18 | 0 | 1 | 4 | 5 | 6 | 1 | 1 | 12 | | | | -- |
| 19 | 0 | 1 | 0 | 1 | 1 | 0 | 0 | 11 | | | | -- |
| 20 | 0 | 1 | 3 | 4 | 5 | 1 | 0 | 11 | | | | -- |

Table S2-9. Extended family with $T$=2 and children are educated at home.

| Data | death toll children | adults | elderly | Total | inf max | immune | inf peak | duration | thresh | temp | Schooling | peaks |
|---|---|---|---|---|---|---|---|---|---|---|---|---|
| 0 | 5 | 2 | 11 | 18 | 19 | 2 | 9 | 22 | 500 | 5 | Home | 1w |
| 1 | 1 | 1 | 3 | 5 | 6 | 1 | 5 | 16 | | | | -- |
| 2 | 2 | 1 | 0 | 3 | 3 | 0 | 6 | 17 | | | | -- |
| 3 | 2 | 1 | 2 | 5 | 6 | 1 | 5 | 16 | | | | -- |
| 4 | 0 | 1 | 0 | 1 | 1 | 0 | 0 | 11 | | | | -- |
| 5 | 1 | 2 | 0 | 3 | 3 | 0 | 7 | 18 | | | | -- |
| 6 | 2 | 4 | 11 | 17 | 14 | 5 | 7 | 30 | | | | 2w |
| 7 | 4 | 3 | 3 | 10 | 8 | 0 | 5 | 33 | | | | 1wd |
| 8 | 1 | 1 | 4 | 6 | 8 | 2 | 6 | 17 | | | | -- |
| 9 | 5 | 5 | 16 | 26 | 27 | 2 | 9 | 24 | | | | 1w |
| 10 | 3 | 1 | 5 | 9 | 9 | 0 | 3 | 14 | | | | -- |

Table S2-10. Extended family with *T*=5 and children are educated at home.

| Data | death toll children | adults | elderly | Total | inf max | Immune | inf peak | duration | thresh | temp | Schooling | peaks |
|---|---|---|---|---|---|---|---|---|---|---|---|---|
| 0 | 0 | 1 | 0 | 1 | 1 | 0 | 0 | 11 | 500 | 10 | Home | -- |
| 1 | 3 | 4 | 10 | 17 | 13 | 6 | 17 | 55 | | | | 1wd |
| 2 | 0 | 1 | 0 | 0 | 1 | 1 | 0 | 11 | | | | -- |
| 3 | 11 | 4 | 15 | 30 | 17 | 5 | 31 | 44 | | | | 2wd |
| 4 | 2 | 2 | 1 | 5 | 6 | 1 | 3 | 14 | | | | -- |
| 5 | 5 | 2 | 8 | 15 | 11 | 2 | 13 | 45 | | | | 1wd |
| 6 | 6 | 5 | 9 | 20 | 19 | 3 | 14 | 28 | | | | 1w |
| 7 | 0 | 0 | 1 | 1 | 1 | 0 | 0 | 11 | | | | -- |
| 8 | 6 | 1 | 15 | 22 | 17 | 6 | 32 | 51 | | | | 1w |
| 9 | 0 | 1 | 0 | 1 | 1 | 0 | 0 | 11 | | | | -- |
| 10 | 2 | 0 | 4 | 6 | 8 | 2 | 8 | 19 | | | | -- |

Table S2-11. Extended family with *T*=10 and children are educated at home.

| Data | death toll children | adults | elderly | Total | inf max | immune | inf peak | duration | thresh | temp | Schooling | peaks |
|---|---|---|---|---|---|---|---|---|---|---|---|---|
| 0 | 0 | 1 | 0 | 0 | 1 | 1 | 0 | 11 | 500 | 15 | Home | -- |
| 1 | 0 | 1 | 0 | 0 | 1 | 1 | 0 | 11 | | | | -- |
| 2 | 2 | 1 | 1 | 3 | 3 | 1 | 11 | 22 | | | | -- |
| 3 | 1 | 0 | 0 | 1 | 1 | 0 | 0 | 11 | | | | -- |
| 4 | 0 | 0 | 1 | 1 | 2 | 1 | 2 | 13 | | | | -- |
| 5 | 0 | 0 | 1 | 0 | 1 | 1 | 0 | 11 | | | | -- |
| 6 | 1 | 1 | 1 | 3 | 2 | 0 | 5 | 25 | | | | -- |
| 7 | 4 | 4 | 7 | 15 | 11 | 3 | 9 | 33 | | | | 1w |
| 8 | 0 | 0 | 1 | 0 | 1 | 1 | 0 | 11 | | | | -- |
| 9 | 3 | 1 | 6 | 10 | 7 | 1 | 9 | 40 | | | | 1w |
| 10 | 0 | 2 | 8 | 10 | 10 | 4 | 20 | 33 | | | | 1w |
| 11 | 0 | 1 | 0 | 1 | 1 | 0 | 0 | 11 | | | | -- |
| 12 | 0 | 1 | 1 | 2 | 2 | 1 | 4 | 21 | | | | -- |
| 13 | 1 | 0 | 0 | 1 | 1 | 0 | 0 | 11 | | | | -- |
| 14 | 1 | 0 | 3 | 4 | 4 | 0 | 9 | 20 | | | | -- |
| 15 | 0 | 1 | 1 | 2 | 2 | 0 | 8 | 19 | | | | -- |
| 16 | 0 | 0 | 0 | 0 | 1 | 1 | 0 | 11 | | | | -- |
| 17 | 1 | 0 | 0 | 1 | 1 | 0 | 0 | 11 | | | | -- |
| 18 | 0 | 1 | 5 | 6 | 7 | 2 | 9 | 21 | | | | -- |
| 19 | 5 | 1 | 10 | 16 | 6 | 1 | 7 | 46 | | | | 2dw |
| 20 | 0 | 0 | 3 | 3 | 3 | 0 | 9 | 20 | | | | -- |
| 21 | 1 | 0 | 0 | 1 | 1 | 0 | 0 | 11 | | | | -- |
| 22 | 5 | 1 | 2 | 8 | 5 | 0 | 23 | 34 | | | | d |
| 23 | 1 | 1 | 2 | 4 | 5 | 2 | 8 | 22 | | | | -- |
| 24 | 1 | 1 | 2 | 4 | 3 | 3 | 5 | 42 | | | | -- |

| | | | | | | | | | | |
|---|---|---|---|---|---|---|---|---|---|---|
| 25 | 4 | 3 | 10 | 17 | 10 | 2 | 16 | 42 | | 1dw |
| 26 | 1 | 0 | 0 | 1 | 1 | 0 | 0 | 11 | | -- |
| 27 | 0 | 0 | 3 | 3 | 3 | 1 | 9 | 27 | | -- |
| 28 | 2 | 0 | 4 | 6 | 6 | 2 | 17 | 28 | | -- |
| 29 | 18 | 8 | 32 | 58 | 24 | 9 | 48 | 61 | | 3dw |
| 30 | 2 | 0 | 0 | 2 | 3 | 1 | 6 | 17 | | -- |
| 31 | 9 | 3 | 13 | 25 | 13 | 3 | 14 | 65 | | 2sw |
| 32 | 1 | 2 | 5 | 8 | 7 | 0 | 9 | 21 | | -- |
| 33 | 0 | 1 | 0 | 1 | 1 | 0 | 0 | 11 | | -- |
| 34 | 0 | 1 | 0 | 1 | 1 | 0 | 0 | 11 | | -- |
| 35 | 0 | 0 | 0 | 0 | 1 | 1 | 0 | 11 | | -- |
| 36 | 0 | 0 | 1 | 1 | 1 | 0 | 0 | 11 | | -- |
| 37 | 1 | 0 | 0 | 1 | 1 | 0 | 0 | 11 | | -- |
| 38 | 0 | 0 | 4 | 4 | 2 | 0 | 7 | 28 | | -- |
| 39 | 0 | 1 | 0 | 1 | 1 | 0 | 0 | 11 | | -- |
| 40 | 0 | 2 | 1 | 3 | 5 | 2 | 7 | 18 | | -- |
| 41 | 0 | 1 | 0 | 1 | 3 | 3 | 9 | 30 | | -- |
| 42 | 1 | 0 | 0 | 1 | 1 | 0 | 0 | 11 | | -- |
| 43 | 6 | 4 | 10 | 20 | 10 | 1 | 15 | 51 | | 1dw |
| 44 | 0 | 1 | 0 | 1 | 1 | 0 | 0 | 11 | | -- |
| 45 | 1 | 0 | 1 | 2 | 1 | 0 | 0 | 21 | | -- |
| 46 | 0 | 1 | 1 | 2 | 2 | 0 | 2 | 13 | | -- |
| 47 | 1 | 0 | 0 | 1 | 1 | 0 | 0 | 11 | | -- |
| 48 | 1 | 3 | 2 | 6 | 5 | 4 | 19 | 49 | | -- |
| 49 | 7 | 2 | 19 | 28 | 14 | 8 | 30 | 55 | | 3dw |
| 50 | 3 | 1 | 0 | 4 | 4 | 0 | 9 | 20 | | -- |

Table S2-12.  Extended family with *T*=15 and children are educated at home.

## Nuclear families

| Data | death toll children | adults | elderly | Total | inf max | immune | inf peak | duration | thresh | temp | Schooling | Peaks |
|---|---|---|---|---|---|---|---|---|---|---|---|---|
| 0 | 603 | 507 | 984 | 2094 | 2372 | 432 | 10 | 41 | 500 | 2 | Public | 1s |
| 1 | 599 | 502 | 1018 | 2119 | 2358 | 463 | 14 | 40 | | | | 1s |
| 2 | 595 | 505 | 1010 | 2110 | 2400 | 461 | 11 | 35 | | | | 1s |
| 3 | 599 | 513 | 988 | 2100 | 2415 | 438 | 12 | 32 | | | | 1s |
| 4 | 603 | 482 | 1038 | 2123 | 2352 | 461 | 11 | 41 | | | | 1s |
| 5 | 591 | 493 | 1003 | 2087 | 2375 | 468 | 13 | 36 | | | | 1s |
| 6 | 591 | 511 | 999 | 2101 | 2377 | 457 | 13 | 35 | | | | 1s |
| 7 | 613 | 515 | 1020 | 2148 | 2340 | 415 | 21 | 46 | | | | 1s |
| 8 | 599 | 510 | 992 | 2101 | 2370 | 448 | 14 | 37 | | | | 1s |
| 9 | 603 | 516 | 1021 | 2140 | 2398 | 436 | 15 | 41 | | | | 1s |
| 10 | 596 | 509 | 991 | 2096 | 2349 | 460 | 20 | 41 | | | | 1s |
| -11 | 588 | 527 | 1011 | 2126 | 2329 | 447 | 14 | 44 | | | | 1s |

Table S2-13. Nuclear family with $T$=2 and children attend the public schools.

| Data | death toll children | adults | elderly | Total | inf max | immune | inf peak | duration | thresh | temp | Schooling | Peaks |
|---|---|---|---|---|---|---|---|---|---|---|---|---|
| 0 | 605 | 429 | 918 | 1952 | 1747 | 401 | 13 | 42 | 500 | 5 | Public | 1s |
| 1 | 593 | 426 | 979 | 1998 | 1826 | 432 | 26 | 52 | | | | 1s |
| 2 | 599 | 427 | 888 | 1914 | 1658 | 427 | 15 | 50 | | | | 1s |
| 3 | 0 | 2 | 6 | 8 | 12 | 4 | 7 | 18 | | | | -- |
| 4 | 596 | 452 | 1025 | 2073 | 1797 | 449 | 25 | 53 | | | | 1s |
| 5 | 594 | 441 | 1038 | 2073 | 1699 | 469 | 14 | 49 | | | | 1s |
| 6 | 0 | 0 | 0 | 0 | 1 | 1 | 0 | 11 | | | | -- |
| 7 | 583 | 424 | 937 | 1944 | 1761 | 448 | 15 | 42 | | | | 1s |
| 8 | 606 | 434 | 975 | 2015 | 1794 | 436 | 14 | 42 | | | | 1s |
| 9 | 0 | 2 | 0 | 2 | 2 | 0 | 6 | 17 | | | | -- |
| 10 | 604 | 425 | 941 | 1970 | 1718 | 428 | 17 | 50 | | | | 1s |

Table S2-14. Nuclear family with *T*=5 and children attend the public schools.

| Data | death toll children | adults | elderly | Total | inf max | immune | inf peak | duration | thresh | temp | Schooling | peaks |
|---|---|---|---|---|---|---|---|---|---|---|---|---|
| 0 | 600 | 293 | 779 | 1672 | 1085 | 359 | 22 | 79 | 500 | 10 | Public | 1s lt |
| 1 | 601 | 286 | 807 | 1694 | 1127 | 355 | 24 | 62 | | | | 1s |
| 2 | 608 | 279 | 776 | 1663 | 1056 | 353 | 25 | 67 | | | | 1s |
| 3 | 601 | 301 | 842 | 1744 | 1033 | 384 | 29 | 70 | | | | 1s |
| 4 | 592 | 314 | 919 | 1825 | 1111 | 368 | 21 | 82 | | | | 1s lt |
| 5 | 608 | 300 | 832 | 1740 | 997 | 339 | 32 | 90 | | | | 1s lt |
| 6 | 594 | 312 | 792 | 1698 | 1099 | 332 | 25 | 70 | | | | 1s lt |
| 7 | 0 | 1 | 0 | 1 | 1 | 0 | 0 | 11 | | | | -- |
| 8 | 581 | 264 | 717 | 1562 | 1109 | 383 | 32 | 82 | | | | 1s ltw |
| 9 | 604 | 300 | 781 | 1685 | 1139 | 352 | 22 | 83 | | | | 1s lt |
| 10 | 598 | 293 | 808 | 1699 | 1087 | 337 | 24 | 79 | | | | 1s lt |

Table S2-15. Nuclear family with *T*=10 and children attend the public schools.

| Data | death toll | | | | inf max | immune | inf peak | duration | thresh | temp | Schooling | peaks |
|---|---|---|---|---|---|---|---|---|---|---|---|---|
| | children | adults | elderly | Total | | | | | | | | |
| 0 | 0 | 1 | 0 | 1 | 1 | 0 | 0 | 11 | 500 | 15 | Public | -- |
| 1 | 587 | 263 | 847 | 1697 | 769 | 346 | 50 | 101 | | | | 1w |
| 2 | 0 | 0 | 15 | 15 | 14 | 2 | 10 | 24 | | | | 1wd |
| 3 | 597 | 250 | 833 | 1680 | 877 | 320 | 38 | 86 | | | | 1w |
| 4 | 589 | 236 | 817 | 1642 | 862 | 356 | 40 | 101 | | | | 1w |
| 5 | 0 | 1 | 0 | 1 | 1 | 0 | 0 | 11 | | | | -- |
| 6 | 0 | 2 | 17 | 19 | 18 | 2 | 9 | 28 | | | | 1w |
| 7 | 583 | 266 | 889 | 1738 | 724 | 333 | 27 | 100 | | | | 1w |
| 8 | 0 | 0 | 0 | 0 | 1 | 1 | 0 | 11 | | | | -- |
| 9 | 604 | 248 | 795 | 1647 | 871 | 307 | 36 | 96 | | | | 1w |
| 10 | 0 | 0 | 0 | 0 | 1 | 1 | 0 | 11 | | | | -- |

Table S2-16. Nuclear family with *T*=15 and children attend the public schools.

| Data | death toll children | adults | elderly | Total | inf max | immune | inf peak | duration | Thresh | temp | Schooling | peaks |
|---|---|---|---|---|---|---|---|---|---|---|---|---|
| 0 | 3 | 1 | 0 | 4 | 4 | 0 | 1 | 12 | 500 | 1 | Home | --- |
| 1 | 52 | 55 | 144 | 251 | 232 | 56 | 20 | 34 | | | | w |
| 2 | 44 | 54 | 127 | 225 | 182 | 53 | 13 | 32 | | | | w |
| 3 | 0 | 2 | 12 | 14 | 14 | 0 | 6 | 17 | | | | --- |
| 4 | 14 | 12 | 35 | 61 | 69 | 13 | 9 | 22 | | | | w |
| 5 | 0 | 2 | 0 | 2 | 2 | 0 | 0 | 11 | | | | --- |
| 6 | 28 | 25 | 80 | 133 | 104 | 31 | 11 | 34 | | | | w |
| 7 | 0 | 1 | 0 | 1 | 0 | 0 | 0 | 11 | | | | --- |
| 8 | 2 | 2 | 10 | 14 | 14 | 0 | 1 | 12 | | | | --- |
| 9 | 52 | 56 | 138 | 246 | 185 | 49 | 24 | 42 | | | | w |
| 10 | 2 | 2 | 0 | 4 | 4 | 0 | 0 | 11 | | | | --- |

Table S2-17. Nuclear family with $T$=1 and children are educated at home.

| Data | death toll children | adults | elderly | total | inf max | immune | inf peak | duration | thresh | temp | Schooling | peaks |
|---|---|---|---|---|---|---|---|---|---|---|---|---|
| 0 | 151 | 159 | 443 | 753 | 248 | 139 | 78 | 129 | 500 | 2 | Home | 1w |
| 1q | 1 | 2 | 30 | 33 | 42 | 10 | 7 | 21 | | | | 1w |
| 2 | 154 | 159 | 470 | 783 | 273 | 193 | 53 | 84 | | | | 5w |
| 3 | 8 | 10 | 47 | 65 | 62 | 14 | 15 | 26 | | | | 1w |
| 4 | 3 | 3 | 15 | 21 | 22 | 1 | 4 | 15 | | | | 1w |
| 5 | 0 | 0 | 0 | 0 | 1 | 1 | 0 | 11 | | | | --- |
| 6 | 209 | 219 | 568 | 996 | 256 | 229 | 44 | 109 | | | | w |
| 7 | 195 | 214 | 544 | 953 | 193 | 182 | 45 | 105 | | | | w |
| 8 | 0 | 1 | 0 | 1 | 1 | 0 | 0 | 11 | | | | --- |
| 9 | 0 | 2 | 0 | 2 | 2 | 0 | 6 | 17 | | | | --- |
| 10 | 145 | 141 | 441 | 727 | 235 | 153 | 68 | 124 | | | | 3w |
| 1p | 198 | 220 | 608 | 1026 | 399 | 231 | 47 | 95 | | | | 3w |

Table S2-18. Nuclear family with *T*=2 and children are educated at home.

| Data | death toll children | adults | elderly | Total | inf max | immune | inf peak | duration | thresh | temp | Schooling | peaks |
|---|---|---|---|---|---|---|---|---|---|---|---|---|
| 0 | 1 | 0 | 0 | 1 | 2 | 1 | 8 | 19 | 500 | 5 | Home | -- |
| 1 | 154 | 186 | 573 | 913 | 234 | 199 | 104 | 138 | | | | 2wd |
| 2 | 0 | 1 | 0 | 1 | 1 | 0 | 0 | 11 | | | | -- |
| 3 | 3 | 4 | 11 | 18 | 22 | 4 | 7 | 18 | | | | 1w |
| 4 | 160 | 186 | 529 | 875 | 215 | 180 | 44 | 107 | | | | 1w |
| 5 | 1 | 3 | 15 | 19 | 19 | 2 | 4 | 31 | | | | 1s |
| 6 | 2 | 3 | 10 | 15 | 16 | 2 | 8 | 21 | | | | 1w |
| 7 | 218 | 249 | 740 | 1207 | 282 | 261 | 80 | 163 | | | | 1wd r |
| 8 | 3 | 1 | 0 | 4 | 4 | 0 | 6 | 17 | | | | -- |
| 9 | 0 | 1 | 14 | 15 | 18 | 3 | 3 | 14 | | | | 1w |
| 10 | 0 | 1 | 0 | 1 | 1 | 0 | 0 | 11 | | | | -- |

Table S2-19. Nuclear family with *T*=5 and children are educated at home.

| Data | death toll | | | | inf max | immune | inf peak | duration | thresh | temp | Schooling | peaks |
|---|---|---|---|---|---|---|---|---|---|---|---|---|
| | children | adults | elderly | total | | | | | | | | |
| 0 | 68 | 108 | 334 | 510 | 97 | 106 | 81 | 144 | 500 | 10 | Home | 5wd |
| 1 | 65 | 84 | 294 | 443 | 148 | 94 | 51 | 105 | | | | 2wd 1s |
| 2 | 5 | 4 | 13 | 22 | 15 | 4 | 12 | 64 | | | | 1wd |
| 3 | 26 | 21 | 67 | 114 | 41 | 26 | 14 | 76 | | | | 3wd |
| 4 | 11 | 22 | 84 | 117 | 51 | 19 | 45 | 74 | | | | 2sd 1wd |
| 5 | 57 | 94 | 309 | 460 | 118 | 93 | 50 | 110 | | | | 3w |
| 6 | 0 | 1 | 0 | 1 | 1 | 0 | 0 | 11 | | | | -- |
| 7 | 1 | 0 | 0 | 1 | 2 | 1 | 9 | 20 | | | | -- |
| 8 | 2 | 4 | 30 | 36 | 22 | 9 | 9 | 36 | | | | 2d |
| 9 | 85 | 110 | 335 | 530 | 105 | 104 | 88 | 137 | | | | d |
| 10 | 2 | 5 | 38 | 45 | 36 | 7 | 5 | 64 | | | | 2: 1s 1w |

Table S2-20. Nuclear family with *T*=10 and children are educated at home.

| Data | death toll children | adults | elderly | total | inf max | immune | inf peak | duration | thresh | temp | Schooling | peaks |
|---|---|---|---|---|---|---|---|---|---|---|---|---|
| 0 | 2 | 0 | 0 | 2 | 1 | 0 | 0 | 21 | 500 | 15 | Home | -- |
| 1 | 0 | 1 | 0 | 1 | 1 | 0 | 0 | 11 | | | | -- |
| 2 | 0 | 0 | 0 | 0 | 1 | 1 | 0 | 11 | | | | -- |
| 3 | 5 | 14 | 63 | 82 | 25 | 25 | 76 | 94 | | | | 3d |
| 4 | 8 | 6 | 16 | 30 | 28 | 5 | 14 | 31 | | | | 1s |
| 5 | 2 | 1 | 0 | 3 | 3 | 0 | 8 | 19 | | | | -- |
| 6 | 0 | 5 | 12 | 17 | 17 | 6 | 15 | 29 | | | | 1s |
| 7 | 84 | 113 | 501 | 698 | 168 | 145 | 95 | 175 | | | | 3d |
| 8 | 13 | 12 | 20 | 45 | 25 | 6 | 42 | 60 | | | | 2d |
| 9 | 4 | 3 | 9 | 16 | 17 | 3 | 9 | 34 | | | | 1w |
| 10 | 118 | 159 | 714 | 991 | 171 | 178 | 99 | 179 | | | | d |
| 11 | 2 | 1 | 13 | 16 | 13 | 2 | 15 | 28 | | | | 1w |
| 12 | 1 | 0 | 0 | 1 | 1 | 0 | 0 | 11 | | | | -- |
| 13 | 0 | 1 | 0 | 1 | 1 | 0 | 0 | 11 | | | | -- |
| 14 | 5 | 3 | 11 | 19 | 13 | 6 | 9 | 37 | | | | 1w |
| 15 | 0 | 1 | 0 | 1 | 1 | 0 | 0 | 11 | | | | -- |
| 16 | 7 | 17 | 83 | 107 | 63 | 24 | 41 | 62 | | | | lt 1s |
| 17 | 0 | 1 | 0 | 1 | 1 | 0 | 0 | 11 | | | | -- |
| 18 | 2 | 0 | 0 | 2 | 3 | 1 | 7 | 18 | | | | -- |
| 19 | 0 | 0 | 0 | 0 | 1 | 1 | 0 | 11 | | | | -- |
| 20 | 3 | 9 | 39 | 51 | 18 | 5 | 36 | 54 | | | | 2wd |
| 21 | 0 | 0 | 0 | 0 | 1 | 1 | 0 | 11 | | | | -- |
| 22 | 0 | 4 | 12 | 16 | 12 | 2 | 18 | 32 | | | | 1w |
| 23 | 1 | 4 | 24 | 29 | 33 | 8 | 20 | 33 | | | | 1s |
| 24 | 0 | 1 | 0 | 1 | 1 | 0 | 0 | 11 | | | | -- |

| | | | | | | | | | | | |
|---|---|---|---|---|---|---|---|---|---|---|---|
| 25 | 15 | 21 | 71 | 107 | 43 | 25 | 41 | 80 | | | 1w,2s |
| 26 | 0 | 0 | 2 | 2 | 2 | 0 | 4 | 15 | | | -- |
| 27 | 1 | 1 | 0 | 2 | 0 | 2 | 0 | 11 | | | -- |
| 28 | 0 | 0 | 7 | 7 | 3 | 4 | 9 | 42 | | | -- |
| 29 | 3 | 2 | 8 | 13 | 9 | 3 | 18 | 34 | | | 1w |
| 30 | 60 | 102 | 457 | 619 | 123 | 128 | 119 | 192 | | | 1d,1w,1d |
| 31 | 3 | 1 | 0 | 4 | 3 | 0 | 5 | 26 | | | -- |
| 32 | 20 | 21 | 64 | 105 | 39 | 29 | 13 | 72 | | | 1s, 1d |
| 33 | 18 | 30 | 113 | 161 | 97 | 36 | 34 | 62 | | | 1d,1s |
| 34 | 5 | 7 | 27 | 39 | 24 | 6 | 50 | 69 | | | 1d, 1s |
| 35 | 0 | 1 | 0 | 1 | 1 | 0 | 0 | 11 | | | -- |
| 36 | 18 | 16 | 55 | 89 | 31 | 17 | 14 | 71 | | | 2wd |
| 37 | 4 | 2 | 0 | 6 | 5 | 0 | 10 | 21 | | | -- |
| 38 | 26 | 17 | 63 | 106 | 47 | 17 | 34 | 112 | | | 3s, 2d |
| 39 | 0 | 1 | 0 | 1 | 1 | 0 | 0 | 11 | | | -- |
| 40 | 2 | 1 | 0 | 3 | 2 | 1 | 9 | 38 | | | -- |
| 41 | 4 | 2 | 0 | 6 | 4 | 1 | 8 | 31 | | | -- |
| 42 | 11 | 13 | 37 | 61 | 20 | 5 | 68 | 83 | | | 3wd |
| 43 | 1 | 1 | 10 | 12 | 10 | 1 | 9 | 36 | | | 1wd |
| 44 | 54 | 77 | 295 | 426 | 105 | 85 | 70 | 164 | | | 2wd |
| 45 | 74 | 89 | 351 | 514 | 85 | 116 | 210 | 374 | | | 1w lt 3w |
| 46 | 49 | 78 | 299 | 426 | 72 | 84 | 166 | 222 | | | ?? W |
| 47 | 0 | 0 | 0 | 0 | 0 | 1 | 0 | 11 | | | -- |
| 48 | 38 | 42 | 187 | 267 | 64 | 52 | 57 | 140 | | | 5w |
| 49 | 3 | 5 | 12 | 20 | 14 | 2 | 18 | 39 | | | 1w |
| 50 | 0 | 1 | 0 | 1 | 1 | 0 | 0 | 11 | | | -- |
| 51 | 0 | 1 | 0 | 1 | 1 | 0 | 0 | 11 | | | -- |

Table S2-21.  Nuclear family with *T*=15 and children are educated at home.
